# Homologous recombination proficient subtypes of high-grade serous ovarian cancer: treatment options for a poor prognosis group

**DOI:** 10.3389/fonc.2024.1387281

**Published:** 2024-06-04

**Authors:** Nadja Stiegeler, Dale W. Garsed, George Au-Yeung, David D. L. Bowtell, Viola Heinzelmann-Schwarz, Tibor A. Zwimpfer

**Affiliations:** ^1^ Medical Faculty, University of Basel, Basel, Switzerland; ^2^ Cancer Research, Peter MacCallum Cancer Centre, Melbourne, VIC, Australia; ^3^ Sir Peter MacCallum Department of Oncology, The University of Melbourne, Melbourne, VIC, Australia; ^4^ Department of Gynecological Oncology, University Hospital Basel, Basel, Switzerland

**Keywords:** ovarian cancer, homologous recombination proficiency, treatment resistance, PARP inhibitor, CDK inhibitor, PI3K inhibitor, antibody drug conjugate (ADC), vaccine

## Abstract

Approximately 50% of tubo-ovarian high-grade serous carcinomas (HGSCs) have functional homologous recombination-mediated (HR) DNA repair, so-called HR-proficient tumors, which are often associated with primary platinum resistance (relapse within six months after completion of first-line therapy), minimal benefit from poly(ADP‐ribose) polymerase (PARP) inhibitors, and shorter survival. HR-proficient tumors comprise multiple molecular subtypes including cases with *CCNE1* amplification, *AKT2* amplification or *CDK12* alteration, and are often characterized as “cold” tumors with fewer infiltrating lymphocytes and decreased expression of PD-1/PD-L1. Several new treatment approaches aim to manipulate these negative prognostic features and render HR-proficient tumors more susceptible to treatment. Alterations in multiple different molecules and pathways in the DNA damage response are driving new drug development to target HR-proficient cancer cells, such as inhibitors of the CDK or P13K/AKT pathways, as well as ATR inhibitors. Treatment combinations with chemotherapy or PARP inhibitors and agents targeting DNA replication stress have shown promising preclinical and clinical results. New approaches in immunotherapy are also being explored, including vaccines or antibody drug conjugates. Many approaches are still in the early stages of development and further clinical trials will determine their clinical relevance. There is a need to include HR-proficient tumors in ovarian cancer trials and to analyze them in a more targeted manner to provide further evidence for their specific therapy, as this will be crucial in improving the overall prognosis of HGSC and ovarian cancer in general.

## Graphical Abstract

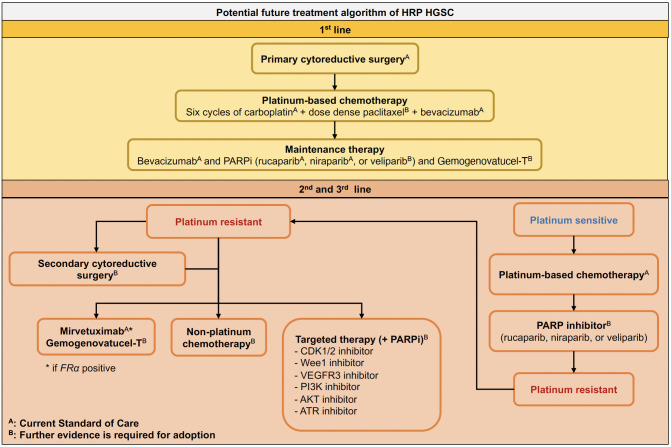

## Introduction

Advanced tubo-ovarian high-grade serous carcinoma (HGSC) accounts for a majority of the disease burden and deaths from ovarian cancer (70–80%) due to its typical late presentation and high 5-year recurrence rate of 75% (1–3). Primary cytoreductive surgery followed by platinum- and taxane-based chemotherapy or neoadjuvant platinum- and taxane-based chemotherapy (NACT) followed by interval cytoreductive surgery is the standard treatment for HGSC (1–9). Most HGSC initially respond well to chemotherapy. However, the majority of patients will experience relapse with treatment resistant disease, particularly those without *BRCA* mutations and without homologous recombination deficiency (HRD) (10–13). Although there has been limited improvement in the 5-year survival rate of most patients over the past three decades (4, 8, 14–16), the introduction of poly(ADP-ribose) polymerase inhibitors (PARPis) as maintenance therapy in HGSC has had a profound impact leading to significant improvements in progression-free survival (PFS) and demonstrating a trend towards improved overall survival (OS), particularly in patients with *BRCA1* or *BRCA2 (BRCA)* mutations and HRD (1, 3, 13, 17–29).

HRD refers to a loss of homologous recombination-mediated DNA repair (HRR), which is a pathway responsible for the high-fidelity repair of double-stranded DNA breaks that restores the original DNA sequence at the site of damage. HRD contributes to genomic instability and consequently intact HRR plays a role in preventing malignant transformation (30, 31). HRD is caused by inherited or somatic loss of function genetic alterations in well-known driver genes such as *BRCA1* and *BRCA2*, but also by mutations or methylation of other HRR related genes and potentially other currently undefined mechanisms (32). Patients with HRD HGSC are more likely to benefit from a favorable chemotherapy response, maintenance treatment with PARPis and consequently a longer OS (1, 33–38). However, ~50% of HGSC are HR-proficient (HRP), an established poor prognostic marker associated with primary platinum and PARPi resistance and shorter survival times (36, 39, 40). Platinum-resistant ovarian cancer is defined as disease that relapses within six months of completing first-line treatment, and the probability of a response to platinum re-treatment is less than 10% (33, 41, 42). In fact, HGSC can also progress from HRD to become at least partially HRP by reversion of HR gene alterations through secondary genetic or epigenetic events (43–45). This acquired HR-proficiency is one of the most well described mechanisms of acquired treatment resistance and consequently a major clinical challenge.

HRD status in ovarian cancer is usually inferred by measurement of *BRCA* mutation status and/or the extent of cancer genome scarring associated with loss of HRR genes. Methodologies that assess HRD typically measure the extent of telomeric allelic imbalance, loss of heterozygosity, and large-scale transitions (31, 46). However, these scores are based on permanent genomic scars, thus failing to reflect the current HRD status in the case of HRR restoration (47). An alternative is a dynamic assessment of HR status using functional assays in *ex-vivo* cultures (46). Immunofluorescence microscopy can be used to measure the presence of RAD51 formed molecular complexes which accumulate at sites of double-stranded DNA breaks in HRP cells. By contrast, HRD cells are unable to form RAD51 formed molecular complexes and their absence thus provides a functional indication of a defect in the HR pathway (48, 49). However, such RAD51 assays are yet to be clinically validated. Additionally, resistance to PARPis may be driven by RAD51-independent mechanisms and consequently cannot be detected by RAD51 assays (50, 51). Current HRD tests vary in the number and type of mutational features assessed, and the optimal thresholds to classify samples as HRD or HRP are not yet well defined. Variation in assays should be considered when evaluating the overall value of such assays in providing prognostic and predictive information.

The heterogeneity of HGSC, including multiple molecular subtypes even within the HRP subgroup, poses a substantial challenge to proper prognostication and clinical management (3, 33, 36, 43, 52, 53). Treatment options for patients with platinum-resistant, non-HRD HGSC are scarce, and the goal of treatment is strongly focused on symptom control and palliation, delaying time to symptomatic progression and improving quality of life (33, 53–57). To date, apart from the antibody-drug conjugate (ADC) mirvetuximab (Elahere^®^), few treatments in addition to cytoreductive surgery and platinum- and taxane-based chemotherapy have shown a survival benefit in this poor prognosis group (3, 33, 42, 53–57).

Recent novel approaches to treat ovarian cancer has largely benefitted patients with HRD HGSC, with or without *BRCA*-alterations (1, 3, 13, 17–29, 58). Further progress in the treatment of HGSC requires approaches that benefit patients with HRP disease, who currently have limited treatment options other than surgery. Here we summarize recent clinical and molecular findings in HRP HGSC and provide an insight into ongoing trials of new potential treatment options.

## Characteristics of patients with HR-proficient HGSC

### Clinicopathological

Variation in outcomes between patients with HGSC is in part determined by the molecular characteristics of the tumor, with HR-status as one of the important determinants (**Table 1**). Patients with HRP tumors have an older median age at diagnosis compared to patients with HRD tumors (10, 11, 20, 36). A retrospective analysis of 352 patients showed that HRP tumors required a higher number of cycles of NACT to be considered for interval cytoreductive surgery compared to those with germline *BRCA* mutations and other defects conferring HRD, and less complete gross resection (R0) could be achieved (11). While complete resection in primary and interval cytoreductive surgery remains one of the strongest prognostic features in ovarian cancer (2, 3, 62, 63), the higher number of chemotherapy cycles and lower R0 rate also reflect an inherently resistant tumor (18, 22–24, 26, 33).

**Table 1:** Clinicopathological characteristics for Non-HRD/HRP versus HRD HGSC.

	Non-HRD/HRP	HRD
**Median age (years) (10, 11, 20, 36)**	63–64	Germline *BRCA*+: 54–58.5 Somatic *BRCA*/HRD+: 58–62
**Frequency (%) (10, 11, 36)**	∼50%	∼50%
**Non-serous histology subtypes (11)**	20%	Germline *BRCA*+: 6% Somatic *BRCA*/HRD+: 0%
**Molecular characteristics (59–61)**	*CCNE1*-amplification *AKT2*-amplification Whole genome duplication	*BRCA1* and *BRCA2* or other HR genes (*BRIP1*, *PALB2*, *RAD51C, RAD51D*)
**Median NACT cycles required (10, 11)**	4	Germline *BRCA*+: 3 Somatic *BRCA*/HRD+: 3
**Rate of complete gross resection (11)**	60%	Germline *BRCA*+: 83% Somatic *BRCA*/HRD+: 77%
**Median progression-free survival (months) (10–13)**	5.4–16.9	Germline *BRCA*+: 23.5–25 Somatic *BRCA*/HRD+: 20.2–25.2
**Median overall survival (months) (11, 17)**	40.4–42.3	Germline *BRCA*+: 68.8 Somatic *BRCA*/HRD+: 69.2

Adapted from (10–13, 17, 20, 36, 59–61).

NACT, Neoadjuvant Chemotherapy; HRP, Homologous recombination proficient; HRD, Homologous recombination deficien; HR, Homologous recombination.

### Genomic characteristics

Extensive genomic and transcriptomic characterization has provided insight into HGSC with HRR pathway inactivation, most commonly caused by genetic or epigenetic alterations in the *BRCA* genes and alterations in other genes, including *BRIP1*, *PALB2*, *RAD51C, or RAD51D*, which encode proteins that are also involved in HR DNA repair (59). By contrast, the molecular drivers of HGSC that have no apparent defects in HR are less well defined (2).

HRP ovarian cancer cells are often characterized by genetic alterations in signaling pathways that contribute to cell cycle dysregulation, such as cyclin E1 (encoded by *CCNE1*) and cyclin dependent kinase (*CDK*) genes (44). Cyclin E1 is an important factor in the G1/S cell cycle transition through its activation of cyclin-dependent kinase 2 (CDK2), allowing the cell to enter the S-phase (64). Besides other cellular mechanisms, limiting the supply of cyclin E1 ensures that the cell remains in the G1 phase by keeping CDK2 inactive until mitogenic signals intervene (65). *CCNE1* expression is dependent on E2F transcription factors that are bound to the retinoblastoma protein (Rb) in an inactivated state when cells are at rest. E2F is released through mitogenic stimuli such as c-MYC which increases the expression of D-type cyclins that in turn combine with CDK4 and CDK6 to phosphorylate and inactivate Rb (65). Furthermore, once activated, the cyclin E1/CDK2 complex is able to phosphorylate Rb and thus upregulate its own expression in the form of a positive feedback loop through the continued release of E2F, independent of mitogenic stimuli (65). Additionally, the cyclin E1/CDK2 complex is an essential component of the chromatin remodeling process required for DNA replication. Overexpression of cyclin E1 increases the speed at which cancer cells transition from G1 to the S phase (66). This can lead to replicative stress, whole genome duplication, and further promote the dysregulation of genes responsible for proliferation and cell survival, which are also associated with resistance to cytotoxic and targeted therapies (67, 68).

*CCNE1* amplification is currently the best characterized driver of HGSC with HR-proficiency. It is important to note, however, that cyclin E1 protein overexpression itself has not been shown to be a predictive biomarker for chemotherapy resistance in epithelial ovarian cancer (EOC), so methods to detect amplification of a gene (e.g. whole-genome sequencing, fluorescence *in situ* hybridization, polymerase chain reaction, single nucleotide polymorphism arrays) are required to identify the *CCNE1* amplified subgroup (69). Approximately 40% of HRP HGSC show an *CCNE1* amplification, which has been shown to be an early event in their development (43, 64). HR pathway gene mutations and *CCNE1* amplification have been shown to be mutually exclusive (44, 60, 65). This suggests that the pathogenesis of HGSC follows at least two distinct pathways, and that *CCNE1*-amplified tumors with cyclin E1 protein overexpression are more likely to be resistant to platinum-based chemotherapy and PARPi due to HR-proficiency (65).

*AKT2* amplification is also a poor prognostic marker in EOC (34, 70, 71) and is associated with *CCNE1* amplification (70). The co-amplification of the serine/threonine-protein kinase *AKT2* and *CCNE1* appears to be explained in part by their proximity on chromosome 19q. Pathway analysis indicates that *CCNE1*-amplified cell lines are dependent on multiple genes within the CDK and AKT pathways, suggesting a specific dependence of *CCNE1*-amplified tumors on AKT activity (70). Consequently, combined CDK2 and AKT inhibition may have synergistic anti-tumor activity against *CCNE1*-amplified tumors and hold promise for clinical development (70). It should be noted that although CDK4/6 inhibitors have been investigated in ovarian cancer (72), it is the CDK2 inhibitor which is likely to be effective (73–76).

*CDK12*-altered HGSC represent a unique subgroup that appear to be HR competent (36). Despite lacking the typical HRD genomic scarring, *CDK12*-altered tumors have a distinct tandem duplication signature and may be more susceptible to chemotherapy and PARPis than other HRP tumors (77). Aside from alterations in *CCNE1, AKT2* and *CDK12*, the majority of HRP HGSC remain poorly defined, and integration of genomic, immune, proteomic and functional data is needed for their complete characterization (78–81).

### Immune profile

Tumor-infiltrating lymphocytes (TILs) are an established prognostic factor in ovarian cancer, regardless of the extent of surgical cytoreduction and chemotherapy (82–84). The presence of CD8+ TILs in the tumor microenvironment is associated with slower tumor progression, prolonged survival and may be essential for immunotherapy response (84–86). HRD tumors have a significantly increased CD8+/CD4+ ratio of TILs and a higher number of peritumoral T cells (44). This is likely due to HRD cells accumulating a high number of somatic mutations, which is predicted to result in the expression of more tumor neoantigens that elicit an adaptive immune response and cytotoxic T cell infiltration. These cells are capable of killing cancer cells (84), and in addition to a more favorable response to chemotherapy, explains the improved survival of patients with *BRCA*-mutated ovarian cancer.

By contrast, HRP tumors are characterized by a non-inflamed or “cold” immune phenotype, with fewer CD3+ and CD8+ TILs as well as decreased expression of PD-1 and PD-L1 (87–89). HRP tumors generally have a lower tumor mutational burden due to having intact DNA repair, which, together with a low TIL density, would predict a poor response to immune checkpoint blockade (84). Therefore, HRP tumors may be poor candidates for targeted immunotherapy with PD-1 and PD-L1 inhibitors as recently shown (90–96). Recent approaches to immunotherapy for cold tumors have focused on restoring inflammation by reprogramming myeloid cells, stromal cells, and vascular epithelial cells (97). Additionally, PARPi, low-dose radiotherapy, epigenetic drugs and anti-angiogenesis therapy may enhance T cell infiltration, suggesting their use in combination with vaccines and redirected T-cells using chimeric antigen receptors or bispecific antibodies (84, 98). However, it should be noted that while T cell infiltration and the expression of PD-L1 and other immune checkpoint markers increases following chemotherapy, unlike primary disease, the extent of infiltration does not correlate with patient survival (99, 100).

## Treatment options for patients with HRP HGSC

### Chemotherapy

Neoadjuvant chemotherapy with interval cytoreductive surgery is currently an alternative for patients with ovarian cancer who have a low chance of initial complete resection and chemosensitive histologic subtypes, or poor health status (1). However, there is a strong correlation between HR-status and response to platinum-based chemotherapy in HGSC; patients with HRP tumors have severely limited responses to chemotherapy, with reported median PFS ranging from 5.4 to 16.9 months (**Table 1**) (10–13). The chemoresistant nature of HRP tumors highlights the potential benefit of favoring the currently recommended option in HGSC (1) of primary debulking surgery followed by adjuvant platinum and taxane-based chemotherapy in these patients.

An ancillary data analysis of the VELIA/GOG-3005 trial focused on paclitaxel dosing schedule and *BRCA* mutation and HR-status (101). Dose-dense (weekly) paclitaxel was compared to a schedule of every three weeks showing an improved PFS with dose-dense paclitaxel in HRP but not in *BRCA*-mutation or HRD tumors. Previous clinical trials of shorter versus longer paclitaxel intervals in ovarian cancer did not evaluate HR status and therefore further studies are needed to confirm this finding (102, 103). Interestingly, it has been shown that paclitaxel suppresses *CDK1* expression via decreased *BRCA1* phosphorylation, thereby reducing HR activity in response to DNA damage and increasing sensitivity to PARPis (104), so this combination represents a potential new treatment strategy that needs to be further investigated in HRP HGSC.

HGSC typically involves extensive peritoneal spread and therefore intraperitoneal chemotherapy and hyperthermic intraperitoneal chemotherapy (HIPEC) have been evaluated in multiple clinical trials. The goal of intraperitoneal chemotherapy is to increase local exposure to the chemotherapeutic agent, and in the case of HIPEC, heated chemotherapy has an additional cytotoxic effect and increases sensitivity to platinum compounds by inducing a transient state of HRD (105). Koole et al. analyzed the effect of HIPEC among patients with ovarian cancer previously enrolled in the phase III OVHIPEC1 trial (105) stratified by *BRCA*-like (HRD) versus non *BRCA*-like (HRP) (106) or *BRCA* mutation status. Although patients with HRD/*BRCA*-wildtype showed a strong benefit in terms of recurrence-free survival (RFS) and a promising trend in OS from HIPEC, this was non-significant in HRP/*BRCA*-wildtype patients and absent in patients with pathogenic *BRCA* mutations, both in terms of RFS and OS (58). It appears that HRP tumors remain resistant to chemotherapy despite hyperthermia. However, there is a lack of long-term survival data for HIPEC, and thus the benefit of this treatment modality remains unclear. The importance of tumor HR status in predicting response and survival following HIPEC may be addressed in ongoing studies (107).

### Poly (ADP-ribose) polymerase inhibitors

Maintenance PARPi therapy after first-line treatment and in the platinum sensitive recurrent setting have become standard treatment options in patients with *BRCA*-mutated and HRD EOC (1, 3). PARP is an enzyme that helps repair DNA damage and PARP inhibition causes an accumulation of single- and double-stranded DNA breaks (108). HRD cells are unable to effectively repair the DNA damage, resulting in an accumulation of chromosomal aberrations and cell death (109). As a maintenance therapy PARPi have led to improved PFS and shown a promising trend towards improved OS in EOC, particularly in patients with *BRCA* mutant and/or HRD tumors (17, 18, 20, 21, 23, 24). While the greatest benefit is seen in HRD cancers, an exploratory analysis of the Phase III PRIMA trial showed improvements in PFS with niraparib versus placebo as first-line maintenance monotherapy, regardless of *BRCA* and HR-status (20). Patients with *BRCA*-wildtype/HRP tumors treated with niraparib who responded to first-line chemotherapy had a median PFS of 8.1 months versus 5.4 months for placebo, with an estimated probability of survival at 24 months of 81% in the niraparib group versus 59% in the placebo group. Therefore, niraparib is clinically approved for use in patients with HRP HGSC, with beneficial effects and a manageable tolerability profile (110, 111).

An exploratory analysis of the VELIA/GOG-3005 trial (27) showed that some patients with HRP ovarian cancer and also poor chemosensitivity may have gained a transient, but non-significant benefit from the addition of the PARPi veliparib to carboplatin-paclitaxel (median PFS 14.7 vs median 6.7 months, HR 0.62, 95% CI 0.37–1.05) (112). The authors of the study hypothesized that veliparib may have induced a chemosensitizing effect on HRP tumors (112, 113). In addition, the Phase III ATHENA-MONO trial demonstrated improved PFS with rucaparib monotherapy compared to placebo in first-line maintenance in patients with newly diagnosed EOC without evidence of HRD (12.1 vs 9.1 months, HR 0.65, 95% CI 0.45–0.95) (13). As a result of such findings, the ESGO-ESMO-ESP consensus guidelines state that niraparib or rucaparib maintenance therapy may be used for patients with HRP HGSC if they have had a complete or partial response to first line chemotherapy or no evidence of disease (1).

### Antiangiogenic treatment

Vascular endothelial growth factor (VEGF) promotes increased vascularity and angiogenesis in response to hypoxic conditions and is a key promoter of tumor growth (114). The anti-angiogenic VEGF monoclonal antibody bevacizumab was the first targeted agent to be approved for use in stage III and IV EOC, showing an improved PFS when used in combination with chemotherapy and as maintenance therapy in the first-line setting, however without OS benefit (115, 116). According to the ESGO-ESMO-ESP consensus guidelines, patients with HRP HGSC may receive platinum-based chemotherapy with bevacizumab followed by bevacizumab maintenance as an alternative to the option of maintenance with rucaparib or niraparib (1). Among other mechanisms of action, bevacizumab exposure may trigger HRD by inducing a hypoxic cellular state that can downregulate HR-related genes such as *BRCA1/2* and *RAD51* (117). In addition, the relative benefit of bevacizumab in EOC has been shown to increase as the disease becomes more platinum resistant (118). A retrospective analysis of 124 patients with platinum-sensitive recurrent ovarian cancer showed extended PFS with bevacizumab in patients with cyclin E1 overexpression (median 16.3 vs 7.1 months, *P*=0.010) (118).

Tumor VEGF secretion has been shown to be at least partially responsible for the development and maintenance of ascites, and the AURELIA trial demonstrated that the addition of bevacizumab to chemotherapy improved ascites control. This beneficial effect is certainly relevant for the HRP group as they are more frequently associated with suboptimal debulking, earlier recurrence and ascites (54). Furthermore, the combination of niraparib and bevacizumab evaluated in the pre-specified subgroup analysis of the AVANOVA trial showed a significant improvement in PFS compared to niraparib alone in the HRP population (HR 0.40, 95% CI 0.19–0.85) (119). The Phase III GOG-218 trial also showed prolonged PFS in patients with no HRR gene mutations who received bevacizumab in addition to standard chemotherapy with carboplatin and paclitaxel (HR 0.71, 95% CI 0.60–0.85, *P* = 0.0001). This benefit was not observed in patients with HRR gene mutations (HR 0.95; 95% CI 0.71–1.26) (120). Therefore, the ESMO guidelines recommend that the decision on bevacizumab versus niraparib maintenance in the HRP population should be based on the patient’s disease and clinical characteristics, the toxicity profile of the two drug classes, the availability of each drug, and national guidelines (3, 121). The ongoing Phase I/II MITO 25 trial (NCT03462212) may provide clearer evidence about potential therapy options by comparing whether the carboplatin-paclitaxel-bevacizumab-rucaparib or carboplatin-paclitaxel-rucaparib arms improve PFS compared to standard carboplatin-paclitaxel-bevacizumab in patients with HRP HGSC.

The inhibition of VEGF receptor-3 (VEGFR3) has been shown to decrease *BRCA1* and *BRCA2* expression in ovarian cancer cells and resulted in increased chemosensitivity (122). The randomized Phase II trial (NCT01116648) showed that the combination of olaparib plus cediranib, a VEGF receptor 1/2/3 inhibitor, significantly improved PFS in relapsed platinum-sensitive EOC compared to olaparib alone (median 17.7 months vs 9 months, *P*=0.005), with the greatest benefit in *BRCA*-wildtype patients (HR 0.32, *P*=0.008) (123). These results suggest that there may be greater synergism between the two agents in HRP tumors, with the response to olaparib in HRP tumors being enhanced by diminished HRR due to VEGFR3 inhibition. However, experimental *in vivo* efficacy data showed that the combination exhibited broad anti-tumor activity independent of HRR and that the combination effect was largely driven by influencing independent mechanisms affecting tumor cells and the tumor microenvironment (124). Clinically, the combination of cediranib and olaparib also showed some activity in the CONVERTO trial, a single-arm Phase IIb study of the two compounds in heavily pretreated, platinum-resistant, non-germline *BRCA*-mutated patients. However, the target objective response rate (ORR) of 20% was not reached (15.6%) and the overall benefit was unclear (OS 13.2 months, 95% CI 9.4–16.4; PFS 5.1 months, 95% CI 3.5–5.5) given it was a single arm study in a disease setting where most patients are expected to progress or die within 12 months (125). A Phase III trial [NCT02446600] in patients with relapsed platinum-sensitive ovarian cancer found that neither the combination of olaparib and cediranib nor olaparib monotherapy improved PFS compared to standard chemotherapy (126). An ongoing Phase II/III trial (NCT02502266) is evaluating cediranib plus olaparib compared to their monotherapies and standard chemotherapy. It remains to be determined if there is a clinical benefit of VEGF receptor inhibitors in treating EOC, particularly in chemoresistant HRP tumors. Future research efforts must focus on identifying other predictive biomarkers for anti-angiogenic therapy, as not all observed responses can be explained by *BRCA* mutation or HR-status.

### Secondary cytoreductive surgery

There have been significant advances in the surgical management of HGSC with improved PFS and OS due to intensification of surgical efforts (62, 127, 128). A multicenter, open-label, randomized, controlled Phase III trial SOC-1 (NCT01611766) demonstrated in 357 patients with platinum-sensitive relapsed ovarian cancer that secondary cytoreductive surgery (SCS) followed by chemotherapy was associated with significantly longer PFS than with chemotherapy alone (median 17.4 vs 11.9 months, HR 0.58, 95% CI 0.45–0.74, *P*<0.0001) (129). Furthermore, the DESKTOP III trial (NCT01166737) analyzed 407 patients with platinum-sensitive recurrent ovarian cancer and showed that SCS followed by chemotherapy leads to a longer OS than chemotherapy alone (median 53.7 vs 46.0 months, HR for death 0.75, 95% CI 0.59–0.96, *P*=0.02). Patients with a complete resection had the most favorable outcome (130). In addition to these two positive studies, in the GOG-0213 trial, which also included patients with platinum-sensitive, recurrent ovarian cancer, SCS followed by chemotherapy did not result in a longer OS than chemotherapy alone (131). There are some differences between the trials that may explain the inconsistent results, such as the additional use of bevacizumab in the DESKTOP III trial (NCT01166737) or the process of selecting patients and centers (130). Therefore, it is important that patients are appropriately counseled about the option of SCS.

The role of surgery in patients with platinum-resistant disease has received increasing attention (132). In fact, patients with HRP tumors may benefit from SCS, similar to patients with low-grade serous ovarian cancer (133). To our knowledge, only three retrospective studies have been published analyzing the role of SCS in patients with platinum-resistant recurrent ovarian cancer. Both Petrillo et al. and Musella et al. showed a prolonged OS after recurrence when SCS was combined with chemotherapy instead of chemotherapy alone (median 32 months vs 8 months, *P*=0.002 and 67 months vs 24 months, *P*=0.035) (134, 135). However, when evaluating these two studies, it is important to consider that they were carried out before the PARPi era and therefore their conclusions must be put into perspective with current treatment options. A recent multicenter retrospective series by Tuninetti et al. in 50 heavily pretreated platinum-resistant ovarian cancer patients showed a statistically significant longer OS in the group of patients who received complete cytoreduction after SCS compared to the very low survival of patients with residual disease (meidan 33 months vs 5 months, HR 4.21, 95% CI 2.07–8.60, *P*=0.001) (136). These retrospective studies did not include stratification by *BRCA* mutation or HR-status, and any discussion of the extent of surgical clearance should also consider how residual disease may be a marker of biology that drives outcome. However, in a recent multicenter retrospective study investigating platinum sensitive recurrent ovarian cancer, SCS was shown to be effective in *BRCA*-wildtype patients, with an improvement in post-recurrence survival (PRS) when complete resection was performed (5-year PRS of 54% vs 42%, *P*=0.048), whereas in *BRCA*-mutated patients, prognosis appears to be related to molecular tumor characteristics rather than tumor resectability (137). A current prospective randomized controlled trial (NCT05633199) is now comparing SCS in platinum-resistant recurrent ovarian cancer and is expected to provide further information on whether and to what extent SCS can be used in the “platinum-resistant” HRP HGSC subgroup. Another advantage of SCS is to opportunistically obtain more comprehensive information on the pathological and molecular characteristics of HRP HGSC and how this may affect tumor evolution and clinical outcome (127). SCS in HGSC warrants further investigation in prospective trials, with particular attention paid to patient *BRCA* and HR-status.

### Immunotherapy and antibody-drug conjugates

Immunotherapy for HGSC has fallen short of expectations, with immune checkpoint inhibitors so far showing limited benefit in ovarian cancer (138–142). However, there are new, potentially promising approaches, including ADCs that deliver a toxic ‘payload’ of chemotherapy directly to cancer cells via a linker attached to an antibody that binds to a specific surface antigen expressed on cancer cells (143). Mirvetuximab is a first-in-class ADC targeting folate receptor α (FRα), a cell surface protein that is commonly overexpressed on ovarian cancer (80–100%) and minimally expressed on normal tissue (144–146). This ADC incorporates the maytansinoid DM4 payload, a potent tubulin-targeting antimitotic agent, and is the first novel agent to demonstrate an OS benefit when used as a single agent compared to chemotherapy alone in platinum-resistant ovarian cancer, as shown in the MIRASOL Phase III clinical trial (NCT04209855) (144). Patients with platinum-resistant, FRα-positive ovarian cancer treated with mirvetuximab (n=227) experienced an OS of 16.46 months (95% CI, 4.46–24.57) vs 12.75 months (95% CI, 10.91–14.36) for the chemotherapy arm (HR 0.67, 95% CI 0.50–0.89, *P*=0.005) and showed fewer Grade 3 or higher adverse events with mirvetuximab than with chemotherapy (41.7% vs 54.1%).

**Figure 1:**
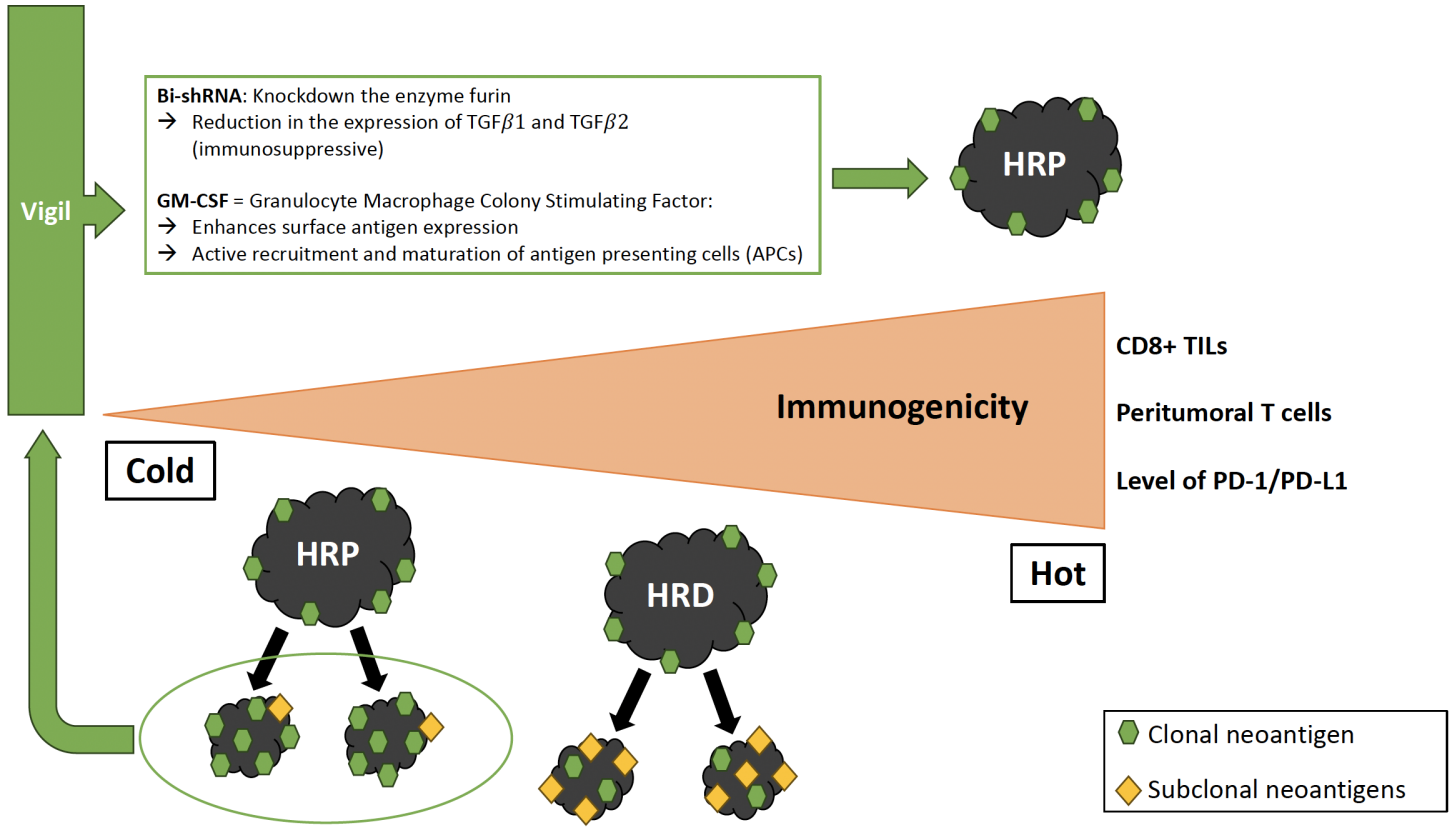
Immune profile of HRP vs. HRD tumors and effect of Vigil. HRP tumors show reduced immunophenotypic markers compared to HRD tumors. Gemogenovatucel-T (Vigil) is a vaccine composed of autologous tumor cells transfected with a plasmid containing GM-CSF and bi-shRNA resulting in a systemic anti-tumor immune response including CD8+ T cell circulation. HRP tumors have a higher proportion of clonal neoantigen expression, which explains the better effect of Vigil on HRP tumors compared to HRD. HRP, Homologous recombination proficient; HRD, Homologous recombination deficient; TILs, Tumor-infiltrating lymphocytes; Bi-shRNA, Bifunctional short hairpin RNA; GM-CSF, Granulocyte/Macrophage Colony Stimulating Factor; TGF, Transforming growth factor; APC, Antigen presenting cell.

Another promising immunotherapy approach is Gemogenovatucel-T (Vigil, formerly known as FANG^®^), the first immunotherapy to demonstrate specific efficacy in the frontline maintenance setting for the HRP population. Vigil is a vaccine composed of autologous tumor cells derived from malignant tissue removed during cytoreductive surgery (147) (**Figure 1**). Tumor cells are transfected with a plasmid containing GM-CSF and bi-shRNA to reduce furin activity, which subsequently downregulates the expression of the immunosuppressive proteins TGF-β1 and TGF-β2 (transforming growth factor β ). This is important because the expression of furin and the resulting immunosuppressive TGF-β isoforms are increased in ovarian tumors compared to normal ovarian tissue (148). Long-term safety of Vigil and evidence of patient benefit have been demonstrated in multiple solid tumors, including advanced ovarian cancer (149, 150). The ongoing Phase IIb VITAL trial (NCT02346747) evaluated the efficacy of Vigil in patients with stage III/IV ovarian cancer. RFS was 11.5 months for patients treated with Vigil versus 8.4 months for patients treated with placebo (HR 0.69, 90% CI 0.44–1.07, *P*=0.078) with an acceptable toxicity profile (151). Although the primary endpoint of RFS was not met, a small subgroup analysis (n=45) showed that RFS and OS was significantly improved with Vigil compared to placebo in HRP patients (HR 0.38 and 0.34, 90% CI 0.2–0.75 and 0.14–0.83, *P*=0.007 and *P*=0.019), while no difference was seen in patients with *BRCA*-mutated disease (151, 152). Vigil increases the expression of cancer-associated neoantigens by upregulating MHC-II and processing by dendritic cells, which enhances the afferent immune response, the initial phase of immune activation characterized by antigen presentation and recognition, resulting in a systemic anti-tumor immune response including CD3+/CD8+ T cell circulation (152). T cells showed to preferentially recognize clonal neoantigens over subclonal neoantigens to target the tumor in lung adenocarcinoma and melanoma (153). HRP tumors are associated with higher clonal neoantigen expression compared to HRD tumors, which therefore contain higher proportions of subclonal neoantigen subpopulations, which may explain why Vigil is more effective on HRP tumors (152). A Phase III trial is planned to validate the efficacy of Vigil compared to bevacizumab and niraparib in the HRP ovarian cancer population (152). It has been suggested that the increased expression of clonal tumor neoantigens and reduced tumor suppressive effect of TGF-β may synergistically enhance the activity of checkpoint inhibitor treatment (84, 154, 155). A prospective, randomized Phase I trial of Vigil plus the immune checkpoint inhibitor atezolizumab in patients with recurrent ovarian cancer explored this approach and showed that the combination was safe, supporting further investigation of this combination, particularly in *BRCA*-wildtype patients (155).

Adoptive cell therapy is another emerging personalized form of immunotherapy in which patients are treated with their *ex vivo* expanded natural TILs, genetically engineered T lymphocytes (CAR T cells) or T-cell receptor (TCR)-engineered T cells, which could offer a potential therapeutic option for patients with cold tumors. To date, CAR T cells that have been tested in clinical trials for HGSC have not yet demonstrated clear benefit (84, 156). While this technology is promising, further development is required to investigate the full potential of T cell engineering and other novel immunotherapy approaches to address the problem of immunologically cold tumors (84).

## Combined targeted therapies

**Figure 2:**
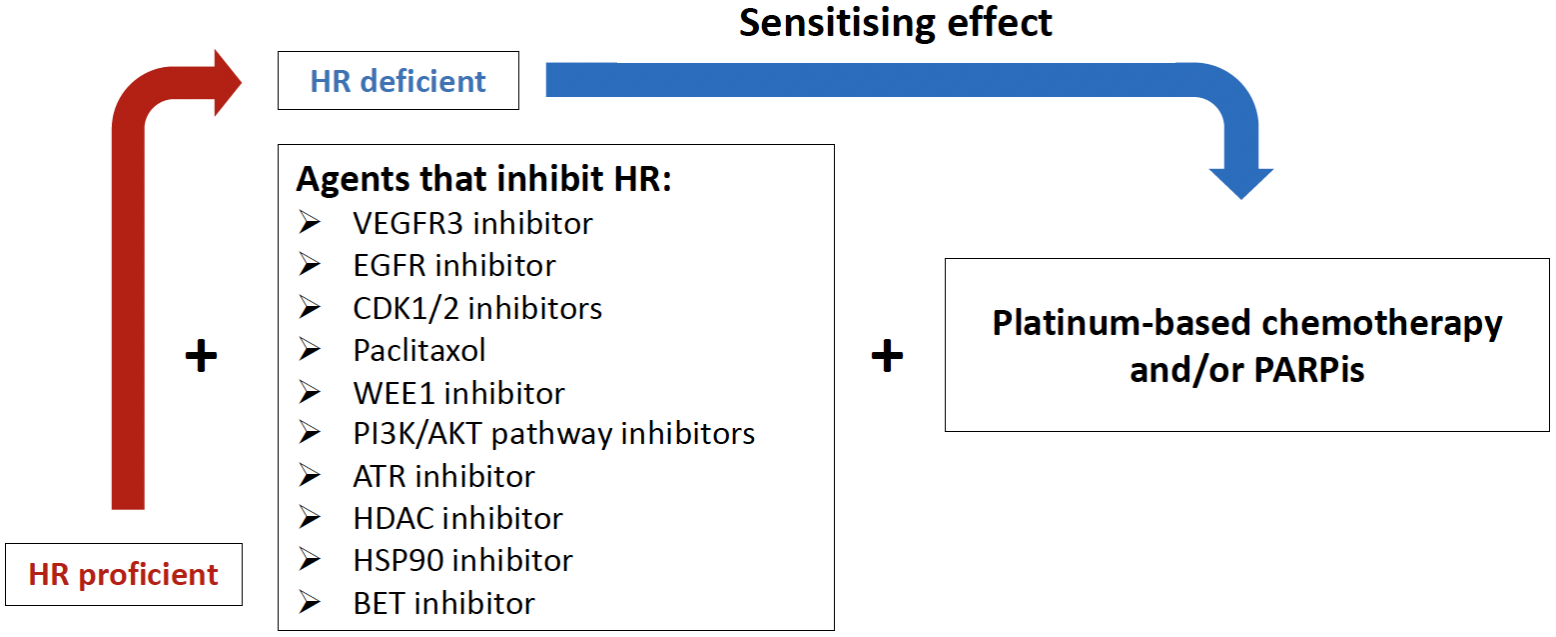
Potential combination therapies to induce homologous recombination deficiency (HRD). VEGFR3i, Vascular endothelial growth factor receptor 3 inhibitor; EGFRi, Epidermal growth factor receptor inhibitor; CDKi, Cyclin-Dependent Kinases inhibitor; WEE1i, Weel-like kinase 1 inhibitor; PI3K, Phosphatidylinositol-3-Kinase and AKT, Serine/threonine protein kinases; ATRi, Ataxia teleangiectasia Rad3-related inhibitor; HDACi, Histone deacetylase inhibitor; HSP90i, Heat shock protein 90 inhibitor; BETi, Brodomain and extraterminal protein family inhibitor.

Rational drug combinations are a potential strategy to prevent or delay the development of resistance and offer the opportunity to improve the therapeutic window by potentially reducing the required drug doses, resulting in fewer side effects (70). Several strategies to selectively disrupt HRR in cancer cells with drugs have been investigated both preclinically and in clinical trials in HGSC or EOC in general, including HRP tumors, and have provided the rationale for new potential therapeutic approaches (**Figure 2**, **Table 2**). Here we review the most promising approaches for HRP tumors that have been or are being investigated in ovarian cancer, including targeting the CDK, P13K/AKT or CHK pathways.

**Table 2:** Clinical trials of potential therapy options for HRP HGSC.

Combinations	Mode of action/possible mechanism of HR suppression	Drug	Phase	Status	Indication	No. of patients	Clinical notes & No. of HRP patients and their evaluation/biomarkers	Study title and references	Clinical- trials.gov
**Chemotherapy + PARPi**	Veliparib may induce a chemo-sensitizing effect.	Carboplatin/Paclitaxel + Veliparib	III	Completed	Newly diagnosed Stage III or IV, high-grade serous, epithelial ovarian, fallopian tube, or primary peritoneal cancer	1140	Improved PFS in HRP tumors (HR 0.76) 372 HRP tumor patients determined by myChoice^®^ assay	VELIA: A Phase III Placebo-Controlled Study of Carboplatin/Paclitaxel With or Without Concurrent and Continuation Maintenance Veliparib in Subjects With Previously Untreated Stages III or IV High-Grade Serous Epithelial Ovarian, Fallopian Tube, or Primary Peritoneal Cancer (27, 101, 112, 113)	NCT02470585
**PARPi**		Niraparib	III	Active, not recruiting	First-line maintenance therapy of advanced ovarian cancer Stage III or IV in complete or partial response to platinum-based chemotherapy	733	Significantly increased PFS (13.8 vs 8.2 months; HR 0.62, p < 0.001) regardless of the presence or absence of HRD, in HRP tumors 8.1 vs 5.4 months (HR 0.68) 249 HRP tumor patients determined by myChoice^®^ assay	PRIMA: A Phase 3, Randomized, Double-Blind-Placebo-Controlled Multicenter Study of Niraparib Maintenance Treatment in Patients With Advanced Ovarian Cancer Following Response on Front-Line Platinum-Based Chemotherapy (20)	NCT02655016
**PARPi + VEGFR3 inhibitor**	Downregulation of *BRCA1/2* gene expression	Olaparib + Cediranib	II/III	Active, not recruiting	Recurrent platinum-resistant or refractory ovarian, fallopian tube or primary peritoneal cancer	562	Cediranib is a VEGFR1, VEGFR2 and VEGFR3 inhibitor Retrospective assessment of HRP tumor patients determined by BROCA HR assay	A Randomized Phase II/III Study of the Combination of Cediranib and Olaparib Compared to Cediranib or Olaparib Alone, or Standard of Care Chemotherapy in Women With Recurrent Platinum-Resistant or Refractory Ovarian, Fallopian Tube, or Primary Peritoneal Cancer (COCOS)	NCT02502266
**PARPi + EGFR inhibitor**	Using synthetic lethality to increase DNA damage	Niraparib + Neratinib	I	Recruiting	Platinum resistant ovarian cancer and other advanced solid tumors	45		iNNOVATE: Phase I/Ib Clinical Trial of Niraparib and Neratinib in Advanced Solid Tumors With an Expansion Cohort in Platinum-resistant Ovarian Cancer	NCT04502602
**Vaccine**	Enhanced expression of clonal tumor neoantigen and reduced tumor suppressor activity of TGF-β	Gemogenovatucel-T (Vigil)	II	Active, not recruiting	Stage IIIb, IIIc or IV high-grade papillary serous/clear cell/endometrioid ovarian, fallopian tube or primary peritoneal cancer	92	A companion clinical Phase II study investigated the combination of Atezolizumab and Vigil in Patients with advanced gynecological cancers (155) 45 HRP tumor patients, retrospective analyzed determined by myChoice^®^ assay	VITAL: A Randomized, Double-Blind, Placebo-Controlled Phase 2 Trial of Vigil Engineered Autologous Tumor Cell immunotherapy in Subjects With Stage IIIb-IV Ovarian Cancer in Clinical Complete Response Following Surgery and Primary Chemotherapy (151)	NCT02346747
**PARPi + immune checkpoint inhibition (+/- bevacizumab)**	Synergistic activity by PARPis activating immune responses	Rucaparib + Nivolumab	III	Active, not recruiting	Advanced high-grade epithelial ovarian, primary peritoneal or fallopian tube cancer who achieved response after cytoreductive surgery and initial platinum-based chemotherapy	1000	Primary outcome is PFS. Primary results of ATHENA-MONO (rucaparib monotherapy) demonstrated improved PFS also in HR proficient patients (12.1 vs 9.1 months; HR 0.65) (13). 44.2% HRP tumor patients, determined by FoundationOne CDx	ATHENA: A multicenter, randomized, double-blind, placebo-controlled Phase III Study in ovarian cancer Patients Evaluating Rucaparib and Nivolumab as Maintenance Treatment Following Response to Front-Line Platinum-Based Chemotherapy (25)	NCT03522246
		Olaparib + Durvalumab	I/II	Active, not recruiting	Patients with advanced solid tumors	264	Preliminary results of the combination in addition with bevacizumab showed an ORR of 75% in HRP patients (157) HRD status determined by FoundationOne CDx	A Phase I/II Study of MEDI4736 (Anti-PD-L1 antibody) in Combination With Olaparib (PARP inhibitor) in Patients With Advanced Solid Tumors	NCT02734004
		Durvalumab + Chemotherapy + Bevacizumab + Olaparib	III	Active, not recruiting	Newly diagnosed advanced ovarian cancer	1407	The HRP subgroup had a consistent PFS effect (HR 0.68, 95%, 0.34–0.86), safety was generally consistent (158) 55% HRP tumor patients determined by myChoice^®^ assay	A Phase III, Double-Blind, Placebo-Controlled, Multicenter Study of Durvalumab in Combination With Chemotherapy and Bevacizumab, Followed by Maintenance Durvalumab, Bevacizumab and Olaparib in Newly Diagnosed Advanced ovarian cancer patients (DUO-O)	NCT03737643
**Selective CDK2 inhibitor**	Rb hypophosphorylation and reduction of CDK2 results in durable control of tumor growth in *CCNE1*-amplified tumors	INX-315	I/II	Recruiting	Advanced cancer, including ovarian cancer with *CCNE1*-ampl.	81	INX-315 is a selective inhibitor of CDK2 Biomarker for HRP tumor patients: *CCNE1*-amplification	A Phase I/II, Open-Label Study to Evaluate the Safety, Tolerability, Pharmacokinetics, and Efficacy of INX-315	NCT05735080
		BLU-222	I/II	Recruiting	Advanced cancer, including ovarian cancer with *CCNE1*-amplification	366	BLU-222 is a selective inhibitor of CDK2 Biomarker for HRP tumor patients: *CCNE1*-amplification	A Phase I/II Study to Evaluate the Safety, Pharmacokinetics, and Efficacy of BLU-222 as a Single Agent and in Combination for Patients With Advanced Solid Tumors	NCT0525416
		PF-07104091	I/II	Recruiting	Advanced or metastatic small cell lung, breast and ovarian cancer	320	PF-07104091 is a selective inhibitor of CDK2, already tested successfully clinically in metastatic breast cancer (75)	Phase 1/IIa Dose Escalation and Expansion Study of PF-07104091 As a Single Agent And in Combination Therapy	NCT04553133
**PARPi + CDK inhibitor**	Inhibition of phosphorylation of *BRCA1*	Veliparib + Dinaciclib	I	Active, not recruiting	Histologically confirmed diagnosis of a solid tumor for which no curative therapy exists	118	Dinaciclib is a multi-CDK inhibitor targeting CDK 1/2/5/9/12	Phase I Trial of ABT-888 and SCH727965 in Patients With Advanced Solid Tumors (159)	NCT01434316
**PARPi + WEE1 inhibitor**	Activation of *CDK1* resulting in cell cycle acceleration and mitotic catastrophe, leading to DNA damage	Olaparib + Adovesertib	II	Recruiting	Ovarian cancer progressed during PARPi therapy	104	Combination shows greater CBR, but the ORR is similar. Better ORR in BRCA wildtype vs BRCA-mutated (39% vs 19%)	EFFORT: Efficacy of AZD1775 in PARP Resistance; a Randomized 2-Arm, Non-Comparative Phase II Study of AZD1775 Alone or AZD1775 and Olaparib in Women With Ovarian Cancer Who Have Progressed During PARP Inhibition (160)	NCT03579316
**PKMYT1 inhibitor + ATR inhibitor**	Downregulation of Myt1 kinase, whose primary role is the negative regulation of *CDK1* in *CCNE1* amplified cells	RP-6306 as monotherapy or with RP-3500	I	Recruiting	Locally advanced or metastatic resistant or refractory solid tumors with next generation sequencing report obtained demonstrating eligible tumor biomarker	180		Phase I of the Safety, Pharmacokinetics, Pharmacodynamics and Preliminary Clinical Activity of RP-6306 Alone or in Combination With RP-3500 in Patients With Advanced Solid Tumors	NCT04855656
**Chemotherapy + PKMYT1 inhibitor**		Gemcitabine + RP-6306	I	Active, not recruiting	Advanced solid tumors	104	Gemcitabine treatment enhances cyclin E-driven DNA replication stress leading to sensitization of cells and tumors to RP-6306	Phase I Study of the PKMYT1 inhibitor RP-6306 in Combination With Gemcitabine for the Treatment of Advanced Solid Tumors (MAGNETIC Study)	NCT05147272
**PARPi + PI3K/AKT pathway inhibitors**	ERK activation/phosphorylation, increased activation of ETS1, and suppression of *BRCA1/2* expression	Olaparib + Alpelisib	II	Active, not recruiting	HGSC with no germline *BCRA* mutation detected	358	Alpelisib and Olaparib versus chemotherapy of physician’s choice	EPIK-O: A Phase III, Multi-center, Randomized (1:1), Open-label, Active-controlled, Study to Assess the Efficacy and Safety of Alpelisib (BYL719) in Combination With Olaparib as Compared to Single Agent Cytotoxic Chemotherapy, in Participants With no Germline BRCA Mutation Detected, Platinum-resistant or Refractory, High-grade Serous Ovarian Cancer (161)	NCT04729387
		Olaparib + Capivasertib	IB/II	Active, not recruiting	Recurrent endometrial, triple-negative breast, and ovarian cancer	159		A Phase Ib Study of the Oral PARP Inhibitor Olaparib With the Oral mTORC1/2 Inhibitor AZD2014 or the Oral AKT Inhibitor AZD5363 for Recurrent Endometrial, Triple Negative Breast, and Ovarian, Primary Peritoneal, or Fallopian Tube Cancer	NCT02208375
**PARPi + ATR inhibitor**	Restriction of the CHK1 pathway and proteins of the HRR pathway	Olaparib + Ceralasertib	II	Recruiting	Recurrent EOC	86		Combination ATR and PARP Inhibitor (CAPRI) trial With AZD 6738 and Olaparib in Recurrent Ovarian Cancer	NCT03462342
**PARPi + HDAC inhibitor**	Downregulation of HR pathway genes	Talazoparib + Belinostat	I	**Recruiting**	**Metastatic breast cancer, metastatic castration resistant prostate cancer, and metastatic ovarian cancer**	25		A Phase I Dose-Escalation Trial of Talazoparib in Combination With Belinostat for Metastatic Breast Cancer, Castration Resistant Prostate Cancer and Ovarian Cancer	NCT04703920
**PARPi + HSP90 inhibitor**	*BRCA1* and other essential HR pathway genes are HSP90 client proteins	Olaparib + HSP90 inhibitor (AT13387)	I	**Completed**	**Solid tumors that are metastatic or cannot be removed by surgery or recurrent ovarian, fallopian tube, primary peritoneal, or triple-negative breast cancer**	28	No unexpected toxicities, prolonged disease stabilization, but no further development of the combination planned	A Phase I Study of PARP Inhibitor Olaparib and HSP90 Inhibitor AT13387 for Treatment of Advanced Solid Tumors With Expansion in Patients With Recurrent Epithelial Ovarian, Fallopian Tube, Peritoneal Cancer or Recurrent Triple-Negative Breast Cancer (162)	NCT02898207
**PARPi + BET inhibitor**	Induction of DNA damage resulting in HRD phenotype	Talazoparib + ZEN003694	II	Recruiting	Recurrent ovarian, fallopian tube or primary peritoneal cancer	33		Phase II Study of a BET Inhibitor, ZEN003694, Combined With a PARP Inhibitor, Talazoparib, in Patients With Recurrent Ovarian Cancer	NCT05071937

PARPi, Poly ADP-ribose polymerase inhibitors; N, Number of patients; HR, Hazard ratio; HRP, Homologous recombination proficiency; HRD, Homologous recombination deficiency; PFS, Progression-free survival; OS, Overall survival; RB, Retinoblastoma protein; EOC, Epithelial ovarian cancer; ORR, Objective response rate; CBR, Clinical benefit rate.

### CDK pathway

**Figure 3:**
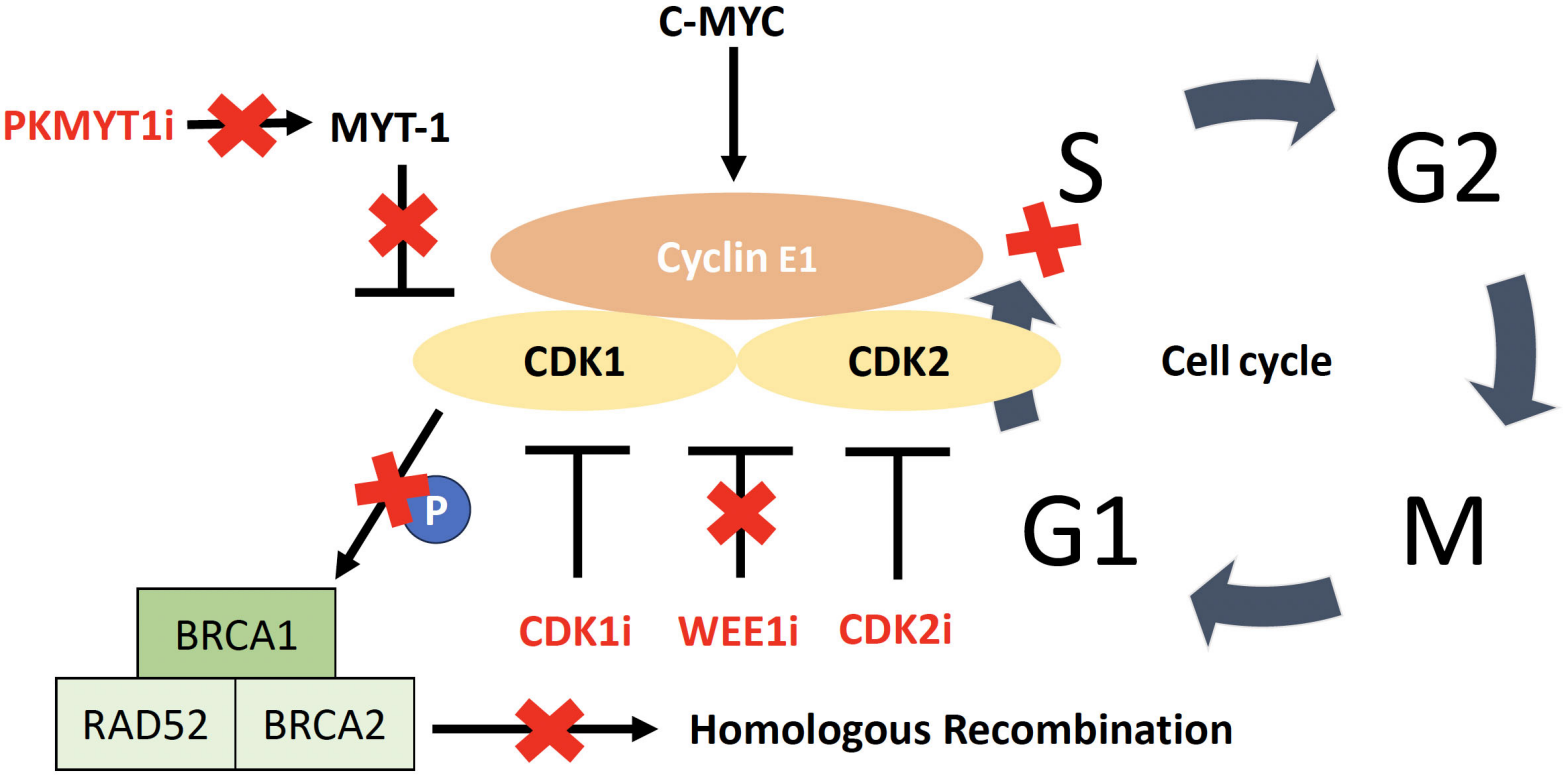
CDK/cyclin E1 complex including targets for therapy. Activation of CDK2 by cyclin E1, allowing the cell to enter the S phase. Overexpression of cyclin E1 increases the rate at which cancer cells transition from G1 to S phase, leading to replicative stress and genomic instability. The WEE1 kinase is involved in regulating cell cycle progression by inhibiting CDK1 and CDK2 and WEE1 inhibition leads to cell cycle acceleration, with early mitotic entry and consequent mitotic catastrophe leading to irreparable DNA damage. CDK1 phosphorylates BRCA1 and CDK1 inhibition impairs the ability of cells to carry out functional DNA repair through homologous recombination. PKMYT1 encodes the protein kinase Myt1, a negative regulator of CDK1.

Approximately 40% of HGSC with HR-proficiency have an amplification of *CCNE1* (64). Cyclins are typically regulatory proteins that modulate the activity of CDKs (65). The CDK pathway offers attractive targets for the treatment of *CCNE1*-amplified tumors due to its role as the kinase partner of cyclin E1 in the activated cyclin E1/CDK complex (65, 163) (**Figure 3**). Cyclin E1 is primarily regulated by *CDK2* in *CCNE1*-amplified tumors, which are selectively dependent on *CDK2* activity (73). Combination therapy with the multi-CDK inhibitor dinaciclib (targets *CDK1/2/5/9*) has shown positive preclinical responses in *CCNE1*-amplified HGSC (164–166), and there is currently an active but not recruiting Phase I trial [NCT01434316] evaluating dinaciclib in combination with the PARPi veliparib in advanced solid tumors. However, a disadvantage of broad-spectrum CDK inhibitors is their high toxicity (167). Recently, more selective CDK2 inhibitors have been investigated (74–76), including promising preclinical results using INX-315, a novel, potent and highly selective CDK2 inhibitor. INX-315 treatment resulted in tumor growth inhibition of *CCNE1*-amplified tumors by promoting retinoblastoma protein hypophosphorylation, inducing cell cycle arrest and delaying the onset of CDK4/6 inhibitor resistance in breast cancer (74). In addition, a recent first-in-human Phase I/IIa study (NCT04553133) of a novel and potent selective CDK2i (PF-07104091) found that it was well tolerated and showed antitumor activity in heavily pretreated metastatic breast cancer patients who had progressed on prior CDK4/6 inhibitors (75). Further development of selective CDK2 inhibitors in Phase I/II clinical trials are ongoing and may be of major importance for HRP HGSC.

Another strategy is to target Weel-like kinase (WEE1), which is highly upregulated in HGSC (108). Its inhibition causes activation of *CDK1* and *CDK2*, resulting in cell cycle acceleration with an early mitotic entry and mitotic catastrophe leading to irreparable DNA damage (121). The multicenter Phase II IGNITE trial [ACTRN12619001185156P] is a non-comparative trial evaluating the WEE1-inhibitor adavosertib in two cohorts of platinum resistant recurrent HGSC (cyclin E1 overexpressed/*CCNE1* amplified and cyclin E1 overexpressed/*CCNE1* non-amplified) and demonstrated an ORR of 53% and a clinical benefit of 61% in an interim analysis of 32 patients in the cyclin E1 overexpressed/*CCNE1* non-amplified cohort (168). *CDK1* is a key cell-cycle regulator and phosphorylates *BRCA1*, which is required for DNA damage-induced checkpoint control through the formation of *BRCA1*-containing foci (169); consequently, inhibition of *CDK1* impairs the ability of cells to functionally repair DNA by HRR (165). Therefore, depletion or inhibition of *CDK1* creates a state of “BRCAness” in transformed cells (170). Results from preclinical studies in other cancer modalities support the effect of WEE1 inhibition on HR, and thus the assumption that WEE1 inhibitors, in combination with a DNA damaging agent, specifically render HRP cell lines more susceptible to treatment (171, 172).

An ongoing Phase II trial (NCT03579316) in recurrent PARPi-resistant EOC (including 98% HGSC) is evaluating the efficacy of the WEE1 inhibitor adavosertib with or without olaparib. The combination showed to have a greater clinical benefit rate than adavosertib alone (89% vs 63%), but the ORR was similar between the two arms (160). Interestingly, exploratory analyses showed a larger benefit of the combination in the *BRCA*-wildtype subgroup compared to the *BRCA*-mutated subgroup (39% vs 19% ORR). Translational analyses are underway to further explore potential predictive biomarkers (160). However, adavosertib use requires consideration of single agent toxicity as well as interactions when used as a drug combination. For example, the use of adavosertib in combination with carboplatin showed an increased incidence of bone marrow suppression, diarrhea, vomiting and fatigue (168, 173). Additionally, adavosertib is metabolized via the enzyme cytochrome P450 3A4 (CYP3A4), which means that patients receiving any co-medications that are strong CYP3A4 inhibitors (for example, antibacterials such as clarithromycin and erythromycin, anticancer agents such as tamoxifen and irinotecan, anti-HIV agents such as ritonavir and delavirdine, or antihypertensives such as dihydro-dralazine and verapamil) (174) would be excluded from clinical trials.

Another promising therapeutic target of the CDK pathway specifically for *CCNE1*-amplified HGSC is *PKMYT1* (68). *PKMYT1* is a kinase encoding the pro-protein kinase Myt1, a negative regulator of *CDK1*, and was identified in a genetic screen of cellular dependencies in *CCNE1* amplified HGSC (68). Inhibition of PKMYT1 results in activation of CDK1, causing unscheduled mitotic entry and genome instability. In contrast, the WEE1inhibitor showed no selectivity towards *CCNE1*-amplified cell lines (175, 176). Ongoing first-in-human clinical trials are evaluating the PKMYT1 inhibitor lunresertib (RP-6306) as monotherapy or in combination with the ataxia-telangiectasia Rad3-related (ATR) inhibitor RP-3500 (NCT04855656) and in combination with gemcitabine (NCT05147272) in advanced solid tumors.

### PI3K/AKT pathway

Phosphatidylinositol 3-kinase (PI3K) activity is stimulated by a wide range of oncogenes and growth factor receptors (177) and the activity of the PI3K pathway is important to the development of drug resistance in a variety of cancer types and treatment settings (178). Inhibition of the PI3K pathway also results in PI3K-mediated downregulation of *BRCA*, accompanied by extracellular signal-regulated kinase (ERK) phosphorylation and subsequent abrogation of HRR (179). Preclinical work in ovarian cancer patient-derived xenograft models has shown that the PI3K inhibitor alpelisib (BYL719) inhibits HRR and consequently sensitizes ovarian cancer models with *de novo* or acquired HR-proficiency to olaparib (180). A Phase I study in 28 patients with EOC (75% HGSC) provided preliminary clinical evidence of the efficacy of the combination of olaparib and alpelisib. An ORR of 33% was seen in patients with *BRCA*-wildtype platinum-resistant EOC compared to an ORR of 3–10% with olaparib or other PARPi monotherapy in the same setting, and with acceptable toxicity (121, 181). Importantly, objective responses to this combination of agents occurred regardless of HR status, as measured by targeted DNA sequencing (181). Further evidence will be provided by the ongoing Phase III EPIK-O/ENGOT-OV61 trial (NCT04729387), which is evaluating the efficacy and safety of alpelisib/olaparib compared to single-agent cytotoxic chemotherapy in patients with platinum-resistant or refractory *BRCA*-wildtype HGSC (161).

The AKT serine/threonine protein kinases (*AKT1*, *AKT2*, *AKT3*) are key downstream mediators of PI3K signaling (182, 183) and in particular, *AKT2* has emerged as a poor prognostic marker and potential target in EOC (34, 70, 71). Drugs targeting AKT have shown activity in breast, endometrial, and ovarian cancer and are currently being investigated in Phase I/II/III trials (183, 184). An active Phase Ib/II trial (NCT02208375) is evaluating the combination of olaparib and the AKT inhibitor capivasertib (AZD5363) in a heavily pretreated cohort of 159 patients, with encouraging clinical activity regardless of the presence of a *BRCA* mutation and despite platinum resistance (183). Further studies are needed to explore the potential of AKT and PI3K inhibitors in combination with PARPi or as monotherapy in HRP HGSC and ovarian cancer in general.

### ATR inhibitors

ATR has a major role in the CHK1 (checkpoint kinase 1) pathway of DNA repair and is a regulator of several proteins in the HRR pathway, including activation of BRCA1, PALB2 and RAD51 (108). The potential for mechanistic synergism between ATR inhibitors (ATRi) and PARPis has been demonstrated in HRD and HRP ovarian cancer cells in preclinical models (108, 185, 186). Acquisition of PARPi resistance was shown to be associated with increased ATR-CHK1 activity, further supporting the potential benefit of combining of PARPis with ATR inhibitors (185). Patient-derived xenograft models of *BRCA*-wildtype and *CCNE1*-amplified platinum-resistant ovarian cancer, which are associated with increased baseline activation of ATR/CHK1, demonstrated tumor reduction and a significant increase in OS when treated with the combination of PARPi and ATRi (185). Based on these preclinical data, an ongoing Phase II clinical trial of ceralasertib (AZD6738) in combination with olaparib was developed and initial results demonstrated the potential of ATRi to overcome PARPi resistance in an HRD HGSC patient population (187).

ATRis are also being investigated as potential monotherapy, and preliminary anti-tumor activity has been demonstrated in heavily pretreated tumors across a range of histologic types and gene alterations (188). Initial results from TRESR, a phase I trial of ATRi monotherapy with camonsertib, support preclinical findings that ATRi may be clinically active in other patient populations beyond those with loss of function of ataxia telangiectasia mutated (ATM) kinase, including those with other gene alterations (e.g., *ARID1A*, *CCNE1*, and *MYC*) or phenotypic (replication) markers (188, 189). The functional assessment of replication stress biomarkers is thought to be a better predictive biomarker for ATRi response than single aberrant genes in ovarian cancer (190). This statement can also be applied to the selective CHK1/2 inhibitor prexasertib, which showed an increased sensitivity to platinum and olaparib in mouse tumor transplantation models and monotherapy efficacy in *BRCA*-wildtype platinum-resistant ovarian cancer (191, 192). To date, however, there is limited data on the safety and anti-tumor activity of CHK inhibitors, and a phase II trial of prexasertib was recently terminated prematurely due to COVID-19 and a shortage of investigational drug supplies (193).

### HDAC inhibitors

The altered expression of HDACs (histone deacetylases) has been associated with resistance to platinum-based chemotherapy and poor prognosis (194) and HDAC inhibition leads to impaired HRR in cancer cells through reduced expression of critical genes such as *BRCA1* and *RAD51* (195, 196). Konstantinopoulos et al. provided a preclinical rationale for the use of HDAC inhibitors (HDACi) to reduce HRR in HRP ovarian cancer, including *CCNE1*-amplified tumors, as a means to enhance PARPi activity (197). This approach has been confirmed by further preclinical studies showing that HDACi such as suberoylanilide hydroamic acid (SAHA), romidepsin, panobinostat and entinostat are synergistic with PARPi in HRP ovarian cancer cells (197, 198). HDACi downregulate genes in the cyclin E/CDK and HR signaling pathways and thus show a synergistic cytotoxic effect in combination with a PARPi (198–200). Based on these preclinical results, there is an ongoing Phase I dose-escalation trial (NCT04703920) of the combination of the PARPi talazoparib and the HDACi belinostat in metastatic ovarian, breast and prostate cancer.

### HSP90

Another attempt to extend the benefit of PARPis to HRP patients is their combination with the heat shock protein 90 (HSP90) inhibitors. HSP90 mediates the maturation, stability and activation of several key proteins involved in DNA repair and HRR, such as CDK1, BRCA1 and BRCA2 (201). Due to its abundant expression, its dependence on adenosine ATP (adenosine triphosphate), and its massive protein interactome, it is an ideal target for pharmacological inhibition (201). Inhibition of HSP90 by ganetespib (STA-9090), a second-generation HSP90 inhibitor, sensitized HRP HGSC cells to talazoparib (201). HSP90 inhibition resulted in downregulation of *BRCA1* and *RAD51*, HRR impairment and increased DNA damage (202). A recent Phase I dose-escalation study showed that the combination of the HSP90i onalespib and olaparib resulted in prolonged disease stabilization, without dose limiting toxicities, in a heavily pretreated patient population with advanced solid tumors (162). Due to limited efficacy as a monotherapy and in other combination studies, further development of onalespib was discontinued (162). However, preclinical and clinical data may support future evaluation of novel combinations of PARPis with other HSP90 inhibitors, such as pimitespib (203). While HSP90 inhibition has the potential to sensitize HRP HGSC to PARPi and other DNA-damaging agents, further clinical research is needed.

### BET inhibitors

The BET (bromodomain and extraterminal) protein family includes BRD4, an epigenetic transcription modulator involved in the expression of proteins that regulate the cell cycle and DNA repair (204). BRD4 has been shown to be a necessary factor for the proliferation and survival of HGSC cells (205). In addition, *BRD4* amplification is mutually exclusive with *BRCA1* and *BRCA2* mutations and tends to co-occur with *CCNE1* amplification in HGSC, so BET inhibition may be particularly promising in the HRP group (38, 206–208). Preclinical studies have shown that BET inhibitors (BETis) suppress the expression of WEE1 and TOPBP1 (DNA Topoisomerase II Binding Protein 1) (209, 210). WEE1 and TOPBP1 play critical roles in cellular processes related to DNA damage response and cell cycle regulation. WEE1 is a protein kinase that regulates the G2/M checkpoint in the cell cycle, controlling entry into mitosis and allowing time for DNA repair (173, 176, 211, 212). TOPBP1 acts as a scaffold protein that coordinates the activation of ATR kinase in response to DNA damage, thereby initiating signaling cascades essential for DNA repair and cell cycle arrest (213). Dysfunction or dysregulation of these proteins can lead to genomic instability and contribute to the development of diseases such as cancer. Additionally, increased BRD4 expression has been identified as a factor contributing to PARPi resistance in HGSC (210). The specific BRD4 inhibitor INCB054329 was able to directly decrease the activity of both *BRCA1* and *RAD51* and induce an HRD phenotype (108, 209). Consequently, in combination with PARPis, a synergistic effect is observed with decreased HR activity, increased DNA damage, and consequently increased tumor cytotoxicity (108, 214). Unfortunately, initial clinical studies involving single agent use of BET inhibitors in various tumor types were disappointing, as preclinical results could not be replicated and resistance to therapy occurred rapidly in some cases (215). Specific evidence in ovarian cancer will be provided by an ongoing Phase II clinical trial (NCT05071937) of the BETi ZEN003694 in combination with the PARPi talazoparib in patients with recurrent ovarian cancer who have progressed on prior PARPi therapy.

## Summary

The HRP HGSC subgroup exhibits complex molecular heterogeneity combined with an immune depleted microenvironment, and these are associated with therapy resistance and a poor prognosis. A subset of these cancers are driven by *CCNE1* amplification and PI3K/AKT alterations that contribute to cell cycle dysregulation and thus these pathways represent promising targets for novel therapeutic approaches. However, a significant subset of HRP HGSC lack *CCNE1* amplification, and the molecular drivers of these cancers are still being defined. Additional studies, including the use of cell lines and potentially the use of existing data from systematic knockdown and knockout genetic screens (216, 217) in the HRP non-*CCNE1* amplified subgroup may define critical dependencies.

A large proportion of HRP HGSC are relatively immune depleted, likely in part due to a reduced mutational burden associated with intact DNA repair. The development of novel immunotherapies to boost the anti-tumor immune response remains a key area of focus for HRP tumors, including personalized approaches to enhance T-cell infiltration with therapeutic vaccines or adoptive cell therapy. Several new combination treatments are under investigation, which aim to sensitize HRP cancers to existing therapies, such as platinum and PARPis, by targeting the HRR pathway and impairing the ability of cells to functionally repair DNA. Antibody drug conjugates also represent a promising class of therapies to increase the potency and specificity of highly potent cytotoxic agents, while reducing toxicity.

These new approaches offer the opportunity to expand the otherwise very limited treatment options for patients with HRP HGSC. Importantly, explicit identification and enrollment of patients with HGSC tumors known to have intact HRR in clinical trials is crucial for the development of effective therapies for this medically underserved group.

## Author contributions

NS: Writing – review & editing, Writing – original draft, Visualization, Methodology, Investigation, Formal analysis, Data curation. DG: Writing – review & editing, Validation, Supervision, Resources, Methodology, Investigation, Funding acquisition. GA-Y: Writing – review & editing, Validation, Supervision, Resources, Project administration, Methodology. DB: Writing – review & editing, Supervision, Resources, Project administration, Methodology. VH-S: Writing – review & editing, Writing – original draft, Supervision, Resources, Project administration, Methodology, Conceptualization. TZ: Writing – review & editing, Writing – original draft, Visualization, Supervision, Resources, Project administration, Methodology, Investigation, Funding acquisition, Formal analysis, Data curation, Conceptualization.

## References

[1] Ledermann JA Matias-Guiu X Amant F Concin N Davidson B Fotopoulou C. ESGO-ESMO-ESP consensus conference recommendations on ovarian cancer: pathology and molecular biology and early, advanced and recurrent disease. Ann Oncol. (2024) 35(3):248–66. doi: 10.1016/j.annonc.2023.11.015 38307807

[2] Bowtell DD Böhm S Ahmed AA Aspuria PJ Bast RC Jr. Beral V. Rethinking ovarian cancer II: reducing mortality from high-grade serous ovarian cancer. . Nat Rev Cancer. (2015) 15(11):668–79. doi: 10.1038/nrc4019 PMC4892184 26493647

[3] González-Martín A Harter P Leary A Lorusso D Miller RE Pothuri B. Newly diagnosed and relapsed epithelial ovarian cancer: ESMO Clinical Practice Guideline for diagnosis, treatment and follow-up. Ann Oncol. (2023) 34(10):833–48. doi: 10.1016/j.annonc.2023.07.011 37597580

[4] Vergote I Tropé CG Amant F Kristensen GB Ehlen T Johnson N. Neoadjuvant chemotherapy primary Surg stage IIIC IV Ovarian cancer. N Engl J Med. (2010) 363(10):943–53. doi: 10.1056/NEJMoa0908806 20818904

[5] Kehoe S Hook J Nankivell M Jayson GC Kitchener H Lopes T. Primary chemotherapy versus primary surgery for newly diagnosed advanced ovarian cancer (CHORUS): an open-label, randomised, controlled, non-inferiority trial. Lancet. (9990) 2015:386. doi: 10.1016/s0140-6736(14)62223-6 26002111

[6] Onda T Satoh T Ogawa G Saito T Kasamatsu T Nakanishi T. Comparison of survival between primary debulking surgery and neoadjuvant chemotherapy for stage III/IV ovarian, tubal and peritoneal cancers in phase III randomised trial. Eur J Cancer. (2020) 130:114–25. doi: 10.1016/j.ejca.2020.02.020 32179446

[7] Fagotti A Ferrandina MG Vizzielli G Pasciuto T Fanfani F Gallotta V. Randomized trial of primary debulking surgery versus neoadjuvant chemotherapy for advanced epithelial ovarian cancer (SCORPION-NCT01461850). . Int J Gynecol Cancer. (2020) 30(11):1657–64. doi: 10.1136/ijgc-2020-001640 33028623

[8] Melamed A Rauh-Hain JA Gockley AA Nitecki R Ramirez PT Hershman DL. Association Between Overall Survival and the Tendency for Cancer Programs to Administer Neoadjuvant Chemotherapy for Patients With Advanced Ovarian Cancer. JAMA Oncol. (2021) 7(12):1782–90. doi: 10.1001/jamaoncol.2021.4252 PMC8485210 34591081

[9] Coleridge SL Bryant A Kehoe S Morrison J. Neoadjuvant chemotherapy before surgery versus surgery followed by chemotherapy for initial treatment in advanced ovarian epithelial cancer. Cochrane Database Syst Rev. (2021) 7(7):Cd005343. doi: 10.1002/14651858.CD005343.pub6 34328210 PMC8406953

[10] Sims T Floyd J Sood A Westin S Fellman B Unke J. Correlation of BRCA and HRD status with clinical and survival outcomes in patients with advanced-stage ovarian cancer in the age of PARPi maintenance therapy (187). Gynecologic Oncol. (2022) 166:S107–S8. doi: 10.1016/S0090-8258(22)01414-7

[11] Sims TT Sood AK Westin SN Fellman BM Unke J Rangel KM. Correlation of HRD status with clinical and survival outcomes in patients with advanced-stage ovarian cancer. J Clin Oncol. (2021) 39(15_suppl):5568. doi: 10.1200/JCO.2021.39.15_suppl.5568

[12] González-Martín A Pothuri B Vergote I Graybill W Lorusso D McCormick CC. Progression-free survival and safety at 3.5years of follow-up: results from the randomised phase 3 PRIMA/ENGOT-OV26/GOG-3012 trial of niraparib maintenance treatment in patients with newly diagnosed ovarian cancer. Eur J Cancer. (2023) 189:112908. doi: 10.1016/j.ejca.2023.04.024 37263896

[13] Monk BJ Parkinson C Lim MC O'Malley DM Oaknin A Wilson MK. A Randomized, Phase III Trial to Evaluate Rucaparib Monotherapy as Maintenance Treatment in Patients With Newly Diagnosed Ovarian Cancer (ATHENA-MONO/GOG-3020/ENGOT-ov45). J Clin Oncol. (2022) 40(34):3952–64. doi: 10.1200/jco.22.01003 PMC9746782 35658487

[14] Vaughan S Coward JI Bast RC Jr. Berchuck A Berek JS Brenton JD. Rethinking ovarian cancer: recommendations for improving outcomes. Nat Rev Cancer. (2011) 11(10):719–25. doi: 10.1038/nrc3144 PMC3380637 21941283

[15] Cabasag CJ Arnold M Rutherford M Ferlay J Bardot A Morgan E. Shifting incidence and survival of epithelial ovarian cancer (1995-2014): A SurvMark-2 study. Int J Cancer. (2023) 152(9):1763–77. doi: 10.1002/ijc.34403 36533660

[16] Network CSE. Ovarian Cancer Survival Rates 2004-2020. Available at: https://seer.cancer.gov/statistics-network/explorer/application.html?site=61&data_type=4&graph_type=2&compareBy=relative_survival_interval&chk_relative_survival_interval_1=1&chk_relative_survival_interval_3=3&chk_relative_survival_interval_5=5&hdn_sex=3&race=1&age_range=1&stage=101&advopt_precision=1&advopt_show_ci=on&hdn_view=0&advopt_show_apc=on&advopt_display=2#resultsRegion0.

[17] Ray-Coquard I Leary A Pignata S Cropet C González-Martín A Marth C. Olaparib plus bevacizumab first-line maintenance in ovarian cancer: final overall survival results from the PAOLA-1/ENGOT-ov25 trial. Ann Oncol. (2023) 34(8):681–92. doi: 10.1016/j.annonc.2023.05.005 37211045

[18] Ray-Coquard I Pautier P Pignata S Pérol D González-Martín A Berger R. Olaparib plus Bevacizumab as First-Line Maintenance in Ovarian Cancer. N Engl J Med. (2019) 381(25):2416–28. doi: 10.1056/NEJMoa1911361 31851799

[19] Moore K Colombo N Scambia G Kim BG Oaknin A Friedlander M. Maintenance Olaparib in Patients with Newly Diagnosed Advanced Ovarian Cancer. N Engl J Med. (2018) 379(26):2495–505. doi: 10.1056/NEJMoa1810858 30345884

[20] González-Martín A Pothuri B Vergote I DePont Christensen R Graybill W Mirza MR. Niraparib in Patients with Newly Diagnosed Advanced Ovarian Cancer. N Engl J Med. (2019) 381(25):2391–402. doi: 10.1056/NEJMoa1910962 31562799

[21] González-Martín A Desauw C Heitz F Cropet C Gargiulo P Berger R. Maintenance olaparib plus bevacizumab in patients with newly diagnosed advanced high-grade ovarian cancer: Main analysis of second progression-free survival in the phase III PAOLA-1/ENGOT-ov25 trial. Eur J Cancer. (2022) 174:221–31. doi: 10.1016/j.ejca.2022.07.022 36067615

[22] DiSilvestro P Banerjee S Colombo N Scambia G Kim BG Oaknin A. Overall Survival With Maintenance Olaparib at a 7-Year Follow-Up in Patients With Newly Diagnosed Advanced Ovarian Cancer and a BRCA Mutation: The SOLO1/GOG 3004 Trial. J Clin Oncol. (2023) 41(3):609–17. doi: 10.1200/jco.22.01549 PMC9870219 36082969

[23] Banerjee S Moore KN Colombo N Scambia G Kim BG Oaknin A. Maintenance olaparib for patients with newly diagnosed advanced ovarian cancer and a BRCA mutation (SOLO1/GOG 3004): 5-year follow-up of a randomised, double-blind, placebo-controlled, phase 3 trial. Lancet Oncol. (2021) 22(12):1721–31. doi: 10.1016/s1470-2045(21)00531-3 34715071

[24] Swisher EM Lin KK Oza AM Scott CL Giordano H Sun J. Rucaparib in relapsed, platinum-sensitive high-grade ovarian carcinoma (ARIEL2 Part 1): an international, multicentre, open-label, phase 2 trial. Lancet Oncol. (2017) 18(1):75–87. doi: 10.1016/s1470-2045(16)30559-9 27908594

[25] Monk BJ Coleman RL Fujiwara K Wilson MK Oza AM Oaknin A. ATHENA (GOG-3020/ENGOT-ov45): a randomized, phase III trial to evaluate rucaparib as monotherapy (ATHENA-MONO) and rucaparib in combination with nivolumab (ATHENA-COMBO) as maintenance treatment following frontline platinum-based chemotherapy in ovarian cancer. Int J Gynecol Cancer. (2021) 31(12):1589–94. doi: 10.1136/ijgc-2021-002933 PMC8666815 34593565

[26] Coleman RL Oza AM Lorusso D Aghajanian C Oaknin A Dean A. Rucaparib maintenance treatment for recurrent ovarian carcinoma after response to platinum therapy (ARIEL3): a randomised, double-blind, placebo-controlled, phase 3 trial. Lancet. (2017) 390(10106):1949–61. doi: 10.1016/s0140-6736(17)32440-6 PMC5901715 28916367

[27] Coleman RL Fleming GF Brady MF Swisher EM Steffensen KD Friedlander M. Veliparib with First-Line Chemotherapy and as Maintenance Therapy in Ovarian Cancer. N Engl J Med. (2019) 381(25):2403–15. doi: 10.1056/NEJMoa1909707 PMC6941439 31562800

[28] Farmer H McCabe N Lord CJ Tutt AN Johnson DA Richardson TB. Targeting the DNA repair defect in BRCA mutant cells as a therapeutic strategy. Nature. (2005) 434(7035):917–21. doi: 10.1038/nature03445 15829967

[29] Tattersall A Ryan N Wiggans AJ Rogozińska E Morrison J. Poly(ADP-ribose) polymerase (PARP) inhibitors for the treatment of ovarian cancer. Cochrane Database Syst Rev. (2022) 2(2):Cd007929. doi: 10.1002/14651858.CD007929.pub4 35170751 PMC8848772

[30] Nguyen L WMM J Van Hoeck A Cuppen E. Pan-cancer landscape of homologous recombination deficiency. Nat Commun. (2020) 11(1):5584. doi: 10.1038/s41467-020-19406-4 33149131 PMC7643118

[31] Marquard AM Eklund AC Joshi T Krzystanek M Favero F Wang ZC. Pan-cancer analysis of genomic scar signatures associated with homologous recombination deficiency suggests novel indications for existing cancer drugs. biomark Res. (2015) 3:9. doi: 10.1186/s40364-015-0033-4 26015868 PMC4443545

[32] Radhakrishnan SK Jette N Lees-Miller SP. Non-homologous end joining: Emerging themes and unanswered questions. DNA Repair. (2014) 17:2–8. doi: 10.1016/j.dnarep.2014.01.009 24582502 PMC4084493

[33] Colombo N Sessa C du Bois A Ledermann J McCluggage WG McNeish I. ESMO-ESGO consensus conference recommendations on ovarian cancer: pathology and molecular biology, early and advanced stages, borderline tumours and recurrent disease†. Ann Oncol. (2019) 30(5):672–705. doi: 10.1093/annonc/mdz062 31046081

[34] Landen CN Molinero L Hamidi H Sehouli J Miller A Moore KN. Influence of Genomic Landscape on Cancer Immunotherapy for Newly Diagnosed Ovarian Cancer: Biomarker Analyses from the IMagyn050 Randomized Clinical Trial. Clin Cancer Res. (2023) 29(9):1698–707. doi: 10.1158/1078-0432.Ccr-22-2032 PMC10150250 36595569

[35] Fong PC Yap TA Boss DS Carden CP Mergui-Roelvink M Gourley C. Poly(ADP)-ribose polymerase inhibition: frequent durable responses in BRCA carrier ovarian cancer correlating with platinum-free interval. J Clin Oncol. (2010) 28(15):2512–9. doi: 10.1200/jco.2009.26.9589 20406929

[36] Garsed DW Pandey A Fereday S Kennedy CJ Takahashi K Alsop K. The genomic and immune landscape of long-term survivors of high-grade serous ovarian cancer. Nat Genet. (2022) 54(12):1853–64. doi: 10.1038/s41588-022-01230-9 PMC10478425 36456881

[37] Pennington KP Walsh T Harrell MI Lee MK Pennil CC Rendi MH. Germline and somatic mutations in homologous recombination genes predict platinum response and survival in ovarian, fallopian tube, and peritoneal carcinomas. Clin Cancer Res. (2014) 20(3):764–75. doi: 10.1158/1078-0432.Ccr-13-2287 PMC3944197 24240112

[38] Bell D Berchuck A Birrer M Chien J Cramer DW Dao F. Integrated genomic analyses of ovarian carcinoma. Nature. (2011) 474(7353):609–15. doi: 10.1038/nature10166 PMC3163504 21720365

[39] Lord CJ Ashworth A. The DNA damage response and cancer therapy. Nature. (2012) 481(7381):287–94. doi: 10.1038/nature10760 22258607

[40] Mukhopadhyay A Elattar A Cerbinskaite A Wilkinson SJ Drew Y Kyle S. Development of a functional assay for homologous recombination status in primary cultures of epithelial ovarian tumor and correlation with sensitivity to poly(ADP-ribose) polymerase inhibitors. Clin Cancer Res. (2010) 16(8):2344–51. doi: 10.1158/1078-0432.Ccr-09-2758 20371688

[41] Ledermann JA. First-line treatment of ovarian cancer: questions and controversies to address. Ther Adv Med Oncol. (2018) 10:1758835918768232. doi: 10.1177/1758835918768232 29662548 PMC5898654

[42] Ledermann JA Raja FA Fotopoulou C Gonzalez-Martin A Colombo N Sessa C. Newly diagnosed and relapsed epithelial ovarian carcinoma: ESMO Clinical Practice Guidelines for diagnosis, treatment and follow-up. Ann Oncol. (2018) 29. doi: 10.1093/annonc/mdy157 30285216

[43] Patch A-M Christie EL Etemadmoghadam D Garsed DW George J Fereday S. Whole–genome characterization of chemoresistant ovarian cancer. Nature. (2015) 521(7553):489–94. doi: 10.1038/nature14410 26017449

[44] Konstantinopoulos PA Ceccaldi R Shapiro GI D'Andrea AD. Homologous Recombination Deficiency: Exploiting the Fundamental Vulnerability of Ovarian Cancer. Cancer Discovery. (2015) 5(11):1137–54. doi: 10.1158/2159-8290.Cd-15-0714 PMC4631624 26463832

[45] Burdett NL Willis MO Pandey A Fereday S Bowtell D Chenevix-Trench G. Small-scale mutations are infrequent as mechanisms of resistance in post-PARP inhibitor tumour samples in high grade serous ovarian cancer. Sci Rep. (2023) 13(1):21884. doi: 10.1038/s41598-023-48153-x 38072854 PMC10711013

[46] Miller RE Leary A Scott CL Serra V Lord CJ Bowtell D. ESMO recommendations on predictive biomarker testing for homologous recombination deficiency and PARP inhibitor benefit in ovarian cancer. Ann Oncol. (2020) 31(12):1606–22. doi: 10.1016/j.annonc.2020.08.2102 33004253

[47] Capoluongo ED Pellegrino B Arenare L Califano D Scambia G Beltrame L. Alternative academic approaches for testing homologous recombination deficiency in ovarian cancer in the MITO16A/MaNGO-OV2 trial. ESMO Open. (2022) 7(5):100585. doi: 10.1016/j.esmoop.2022.100585 36156447 PMC9512829

[48] Castroviejo-Bermejo M Cruz C Llop-Guevara A Gutiérrez-Enríquez S Ducy M Ibrahim YH. A RAD51 assay feasible in routine tumor samples calls PARP inhibitor response beyond BRCA mutation. EMBO Mol Med. (2018) 10(12):e9172. doi: 10.15252/emmm.201809172 30377213 PMC6284440

[49] Guffanti F Alvisi MF Anastasia A Ricci F Chiappa M Llop-Guevara A. Basal expression of RAD51 foci predicts olaparib response in patient-derived ovarian cancer xenografts. Br J Cancer. (2022) 126(1):120–8. doi: 10.1038/s41416-021-01609-1 PMC8727677 34732853

[50] Michelena J Lezaja A Teloni F Schmid T Imhof R Altmeyer M. Analysis of PARP inhibitor toxicity by multidimensional fluorescence microscopy reveals mechanisms of sensitivity and resistance. Nat Commun. (2018) 9(1):2678. doi: 10.1038/s41467-018-05031-9 29992957 PMC6041334

[51] Fuh K Mullen M Blachut B Stover E Konstantinopoulos P Liu J. Homologous recombination deficiency real-time clinical assays, ready or not? Gynecol Oncol. (2020) 159(3):877–86. doi: 10.1016/j.ygyno.2020.08.035 32967790

[52] Le Page C Chung J Rahimi K Köbel M Provencher D Mes-Masson AM. Exploring the Clinical Impact of Predictive Biomarkers in Serous Ovarian Carcinomas. Curr Drug Targets. (2020) 21(10):974–95. doi: 10.2174/1389450120666191016143836 31622218

[53] Davis A Tinker AV Friedlander M. "Platinum resistant" ovarian cancer: what is it, who to treat and how to measure benefit? Gynecol Oncol. (2014) 133(3):624–31. doi: 10.1016/j.ygyno.2014.02.038 24607285

[54] Stockler MR Hilpert F Friedlander M King MT Wenzel L Lee CK. Patient-reported outcome results from the open-label phase III AURELIA trial evaluating bevacizumab-containing therapy for platinum-resistant ovarian cancer. J Clin Oncol. (2014) 32(13):1309–16. doi: 10.1200/jco.2013.51.4240 PMC4876313 24687829

[55] Poveda AM Selle F Hilpert F Reuss A Savarese A Vergote I. Bevacizumab Combined With Weekly Paclitaxel, Pegylated Liposomal Doxorubicin, or Topotecan in Platinum-Resistant Recurrent Ovarian Cancer: Analysis by Chemotherapy Cohort of the Randomized Phase III AURELIA Trial. J Clin Oncol. (2015) 33(32):3836–8. doi: 10.1200/jco.2015.63.1408 26282651

[56] Haun MW Estel S Rücker G Friederich HC Villalobos M Thomas M. Early palliative care for adults with advanced cancer. Cochrane Database Syst Rev. doi: 10.1002/14651858.CD011129.pub2 PMC6481832 28603881

[57] Roncolato FT Joly F O'Connell R Lanceley A Hilpert F Buizen L. Reducing Uncertainty: Predictors of Stopping Chemotherapy Early and Shortened Survival Time in Platinum Resistant/Refractory Ovarian Cancer-The GCIG Symptom Benefit Study. Oncologist. (2017) 22(9):1117–24. doi: 10.1634/theoncologist.2017-0047 PMC5599194 28596446

[58] Koole SN Schouten PC Hauke J Kluin RJC Nederlof P Richters LK. Effect of HIPEC according to HRD/BRCAwt genomic profile in stage III ovarian cancer: Results from the phase III OVHIPEC trial. Int J Cancer. (2022) 151(8):1394–404. doi: 10.1002/ijc.34124 35583992

[59] Lord CJ Ashworth A. BRCAness revisited. Nat Rev Cancer. (2016) 16(2):110–20. doi: 10.1038/nrc.2015.21 26775620

[60] Lord CJ Ashworth A. PARP inhibitors: Synthetic lethality in the clinic. Science. (2017) 355(6330):1152–8. doi: 10.1126/science.aam7344 PMC6175050 28302823

[61] Heeke AL Pishvaian MJ Lynce F Xiu J Brody JR Chen WJ. Prevalence of Homologous Recombination-Related Gene Mutations Across Multiple Cancer Types. JCO Precis Oncol. (2018) 2018:PO.17.00286. doi: 10.1200/po.17.00286 30234181 PMC6139373

[62] du Bois A Reuss A Pujade-Lauraine E Harter P Ray-Coquard I Pfisterer J. Role of surgical outcome as prognostic factor in advanced epithelial ovarian cancer: A combined exploratory analysis of 3 prospectively randomized phase 3 multicenter trials. Cancer. (2009) 115(6):1234–44. doi: 10.1002/cncr.24149 19189349

[63] Kotsopoulos J Zamani N Rosen B McLaughlin JR Risch HA Kim SJ. Impact of germline mutations in cancer-predisposing genes on long-term survival in patients with epithelial ovarian cancer. Br J Cancer. (2022) 127(5):879–85. doi: 10.1038/s41416-022-01840-4 PMC9428139 35710751

[64] Karst AM Jones PM Vena N Ligon AH Liu JF Hirsch MS. Cyclin E1 deregulation occurs early in secretory cell transformation to promote formation of fallopian tube-derived high-grade serous ovarian cancers. Cancer Res. (2014) 74(4):1141–52. doi: 10.1158/0008-5472.Can-13-2247 PMC4517944 24366882

[65] Gorski JW Ueland FR Kolesar JM. CCNE1 Amplification as a Predictive Biomarker of Chemotherapy Resistance in Epithelial Ovarian Cancer. Diagnostics. (2020) 10(5):279. 32380689 10.3390/diagnostics10050279 PMC7277958

[66] Jones RM Mortusewicz O Afzal I Lorvellec M García P Helleday T. Increased replication initiation and conflicts with transcription underlie Cyclin E-induced replication stress. Oncogene. (2013) 32(32):3744–53. doi: 10.1038/onc.2012.387 22945645

[67] Creeden JF Nanavaty NS Einloth KR Gillman CE Stanbery L Hamouda DM. Homologous recombination proficiency in ovarian and breast cancer patients. BMC Cancer. (2021) 21(1):1154. doi: 10.1186/s12885-021-08863-9 34711195 PMC8555001

[68] Gallo D Young JTF Fourtounis J Martino G Álvarez-Quilón A Bernier C. CCNE1 amplification is synthetic lethal with PKMYT1 kinase inhibition. Nature. (2022) 604(7907):749–56. doi: 10.1038/s41586-022-04638-9 PMC9046089 35444283

[69] Sapoznik S Aviel-Ronen S Bahar-Shany K Zadok O Levanon K. CCNE1 expression in high grade serous carcinoma does not correlate with chemoresistance. Oncotarget. (2017) 8(37):62240. 28977941 10.18632/oncotarget.19272 PMC5617501

[70] Au-Yeung G Lang F Azar WJ Mitchell C Jarman KE Lackovic K. Selective Targeting of Cyclin E1-Amplified High-Grade Serous Ovarian Cancer by Cyclin-Dependent Kinase 2 and AKT Inhibition. Clin Cancer Res. (2017) 23(7):1862–74. doi: 10.1158/1078-0432.Ccr-16-0620 PMC5364079 27663592

[71] Foster KI Shaw KRM Jin J Westin SN Yap TA Glassman DM. Clinical implications of tumor-based next-generation sequencing in high-grade epithelial ovarian cancer. Cancer. (2023) 129(11):1672–80. doi: 10.1002/cncr.34724 PMC11949250 36930815

[72] Dall'Acqua A Bartoletti M Masoudi-Khoram N Sorio R Puglisi F Belletti B. Inhibition of CDK4/6 as Therapeutic Approach for Ovarian Cancer Patients: Current Evidences and Future Perspectives. Cancers (Basel). (2021) 13(12):3035. doi: 10.3390/cancers13123035 34204543 PMC8235237

[73] Zhang Z Golomb L Meyerson M. Functional Genomic Analysis of CDK4 and CDK6 Gene Dependency across Human Cancer Cell Lines. Cancer Res. (2022) 82(11):2171–84. doi: 10.1158/0008-5472.Can-21-2428 PMC9618034 35395071

[74] Dietrich C Trub A Ahn A Taylor M Ambani K Chan KT. INX-315, a selective CDK2 inhibitor, induces cell cycle arrest and senescence in solid tumors. Cancer Discovery. (2024) 14(3):446–67. doi: 10.1158/2159-8290.Cd-23-0954 PMC10905675 38047585

[75] Yap TA Elhaddad AM Grisham RN Hamm JT Marks DK Shapiro G. First-in-human phase 1/2a study of a potent and novel CDK2-selective inhibitor PF-07104091 in patients (pts) with advanced solid tumors, enriched for CDK4/6 inhibitor resistant HR+/HER2- breast cancer. J Clin Oncol. 2023 41(16_suppl):3010. doi: 10.1200/JCO.2023.41.16_suppl.3010

[76] Brown V Ramsden P House N Vargas R Guo J Wang R. Abstract 2306: BLU-222, an investigational, potent, and selective CDK2 inhibitor, demonstrated robust antitumor activity in CCNE1-amplified ovarian cancer models. Cancer Res. 82(12_Supplement):2306. doi: 10.1158/1538-7445.Am2022-2306

[77] Dubbury SJ Boutz PL Sharp PA. CDK12 regulates DNA repair genes by suppressing intronic polyadenylation. Nature. (2018) 564(7734):141–5. doi: 10.1038/s41586-018-0758-y PMC6328294 30487607

[78] Zhang H Liu T Zhang Z Payne SH Zhang B McDermott JE. Integrated Proteogenomic Characterization of Human High-Grade Serous Ovarian Cancer. Cell. (2016) 166(3):755–65. doi: 10.1016/j.cell.2016.05.069 PMC4967013 27372738

[79] Dou Y Kawaler EA Cui Zhou D Gritsenko MA Huang C Blumenberg L. Proteogenomic Characterization of Endometrial Carcinoma. Cell. (2020) 180(4):729–48.e26. doi: 10.1016/j.cell.2020.01.026 32059776 PMC7233456

[80] Krug K Jaehnig EJ Satpathy S Blumenberg L Karpova A Anurag M. Proteogenomic Landscape of Breast Cancer Tumorigenesis and Targeted Therapy. Cell. (2020) 183(5):1436–56.e31. doi: 10.1016/j.cell.2020.10.036 33212010 PMC8077737

[81] Vasaikar S Huang C Wang X Petyuk VA Savage SR Wen B. Proteogenomic Analysis of Human Colon Cancer Reveals New Therapeutic Opportunities. Cell. (2019) 177(4):1035–49.e19. doi: 10.1016/j.cell.2019.03.030 31031003 PMC6768830

[82] Sato E Olson SH Ahn J Bundy B Nishikawa H Qian F. Intraepithelial CD8+ tumor-infiltrating lymphocytes and a high CD8+/regulatory T cell ratio are associated with favorable prognosis in ovarian cancer. Proc Natl Acad Sci U.S.A. (2005) 102(51):18538–43. doi: 10.1073/pnas.0509182102 PMC1311741 16344461

[83] Zhang L Conejo-Garcia JR Katsaros D Gimotty PA Massobrio M Regnani G. Intratumoral T cells, recurrence, and survival in epithelial ovarian cancer. N Engl J Med. (2003) 348(3):203–13. doi: 10.1056/NEJMoa020177 12529460

[84] Kandalaft LE Dangaj Laniti D Coukos G. Immunobiology of high-grade serous ovarian cancer: lessons for clinical translation. Nat Rev Cancer. (2022) 22(11):640–56. doi: 10.1038/s41568-022-00503-z 36109621

[85] Consortium OTTA. Dose-Response Association of CD8+ Tumor-Infiltrating Lymphocytes and Survival Time in High-Grade Serous Ovarian Cancer. JAMA Oncol. (2017) 3(12):e173290–e. doi: 10.1001/jamaoncol.2017.3290 PMC5744673 29049607

[86] Garsed DW Alsop K Fereday S Emmanuel C Kennedy CJ Etemadmoghadam D. Homologous Recombination DNA Repair Pathway Disruption and Retinoblastoma Protein Loss Are Associated with Exceptional Survival in High-Grade Serous Ovarian Cancer. Clin Cancer Res. (2018) 24(3):569–80. doi: 10.1158/1078-0432.Ccr-17-1621 29061645

[87] Le DT Durham JN Smith KN Wang H Bartlett BR Aulakh LK. Mismatch repair deficiency predicts response of solid tumors to PD-1 blockade. Science. (2017) 357(6349):409–13. doi: 10.1126/science.aan6733 PMC5576142 28596308

[88] Ciombor KK Goldberg RM. Hypermutated Tumors and Immune Checkpoint Inhibition. Drugs. (2018) 78(2):155–62. doi: 10.1007/s40265-018-0863-0 PMC10184712 29350327

[89] Mouw KW Goldberg MS Konstantinopoulos PA D'Andrea AD. DNA Damage and Repair Biomarkers of Immunotherapy Response. Cancer Discovery. (2017) 7(7):675–93. doi: 10.1158/2159-8290.Cd-17-0226 PMC5659200 28630051

[90] Disis ML Patel MR Pant S Hamilton EP Lockhart AC Kelly K. Avelumab (MSB0010718C; anti-PD-L1) in patients with recurrent/refractory ovarian cancer from the JAVELIN Solid Tumor phase Ib trial: Safety and clinical activity. J Clin Oncol. (2016) 34(15_suppl). doi: 10.1200/JCO.2016.34_suppl.5533

[91] Hamanishi J Mandai M Ikeda T Minami M Kawaguchi A Murayama T. Safety and antitumor activity of Anti-PD-1 antibody, nivolumab, in patients with platinum-resistant ovarian cancer. J Clin Oncol. (2015) 33(34):4015–22. doi: 10.1200/JCO.2015.62.3397 26351349

[92] Varga A Piha-Paul SA Ott PA Mehnert JM Berton-Rigaud D Morosky A. Pembrolizumab in patients (pts) with PD-L1–positive (PD-L1+) advanced ovarian cancer: updated analysis of KEYNOTE-028. J Clin Oncol. (2017) 35(15_suppl). doi: 10.1200/JCO.2017.35.15_suppl.5513 30522700

[93] Ledermann JA Colombo N Oza AM Fujiwara K Birrer MJ Randall LM. Avelumab in combination with and/or following chemotherapy vs chemotherapy alone in patients with previously untreated epithelial ovarian cancer: Results from the phase 3 javelin ovarian 100 trial. . Gynecologic Oncol. (2020) 159:13–4. doi: 10.1016/j.ygyno.2020.06.025

[94] Moore KN Bookman M Sehouli J Miller A Anderson C Scambia G. Atezolizumab, Bevacizumab, and Chemotherapy for Newly Diagnosed Stage III or IV Ovarian Cancer: Placebo-Controlled Randomized Phase III Trial (IMagyn050/GOG 3015/ENGOT-OV39). J Clin Oncol. (2021) 39(17):1842–55. doi: 10.1200/jco.21.00306 PMC8189598 33891472

[95] Matulonis UA Shapira R Santin A Lisyanskaya AS Pignata S Vergote I. Final results from the KEYNOTE-100 trial of pembrolizumab in patients with advanced recurrent ovarian cancer. J Clin Oncol. (2020) 38(15_suppl):6005. doi: 10.1200/JCO.2020.38.15_suppl.6005

[96] Gaillard SL Secord AA Monk B. The role of immune checkpoint inhibition in the treatment of ovarian cancer. Gynecol Oncol Res Pract. (2016) 3:11. doi: 10.1186/s40661-016-0033-6 27904752 PMC5122024

[97] Bruand M Barras D Mina M Ghisoni E Morotti M Lanitis E. Cell-autonomous inflammation of BRCA1-deficient ovarian cancers drives both tumor-intrinsic immunoreactivity and immune resistance *via* STING. Cell Rep. (2021) 36(3):109412. doi: 10.1016/j.celrep.2021.109412 34289354 PMC8371260

[98] Gao Y Wang Y Luo F Chu Y. Optimization of T Cell Redirecting Strategies: Obtaining Inspirations From Natural Process of T Cell Activation. Front Immunol. (2021) 12:664329. doi: 10.3389/fimmu.2021.664329 33981310 PMC8107274

[99] Kim HS Kim JY Lee YJ Kim SH Lee JY Nam EJ. Expression of programmed cell death ligand 1 and immune checkpoint markers in residual tumors after neoadjuvant chemotherapy for advanced high-grade serous ovarian cancer. Gynecol Oncol. (2018) 151(3):414–21. doi: 10.1016/j.ygyno.2018.08.023 30314669

[100] Pearce OMT Delaine-Smith RM Maniati E Nichols S Wang J Böhm S. Deconstruction of a Metastatic Tumor Microenvironment Reveals a Common Matrix Response in Human Cancers. Cancer Discovery. (2018) 8(3):304–19. doi: 10.1158/2159-8290.Cd-17-0284 PMC5837004 29196464

[101] Aghajanian C Swisher EM Okamoto A Steffensen KD Bookman MA Fleming GF. Impact of veliparib, paclitaxel dosing regimen, and germline BRCA status on the primary treatment of serous ovarian cancer – an ancillary data analysis of the VELIA trial. Gynecologic Oncol. (2022) 164(2):278–87. doi: 10.1016/j.ygyno.2021.12.012 PMC9399938 34930617

[102] Katsumata N Yasuda M Isonishi S Takahashi F Michimae H Kimura E. Long-term results of dose-dense paclitaxel and carboplatin versus conventional paclitaxel and carboplatin for treatment of advanced epithelial ovarian, fallopian tube, or primary peritoneal cancer (JGOG 3016): a randomised, controlled, open-label trial. Lancet Oncol. (2013) 14(10):1020–6. doi: 10.1016/S1470-2045(13)70363-2 23948349

[103] Clamp AR James EC McNeish IA Dean A Kim J-W O'Donnell DM. Weekly dose-dense chemotherapy in first-line epithelial ovarian, fallopian tube, or primary peritoneal carcinoma treatment (ICON8): primary progression free survival analysis results from a GCIG phase 3 randomised controlled trial. Lancet. (2019) 394(10214):2084–95. doi: 10.1016/S0140-6736(19)32259-7 PMC6902268 31791688

[104] Yanaihara N Yoshino Y Noguchi D Tabata J Takenaka M Iida Y. Paclitaxel sensitizes homologous recombination-proficient ovarian cancer cells to PARP inhibitor via the CDK1/BRCA1 pathway. Gynecologic Oncol. (2023) 168:83–91. doi: 10.1016/j.ygyno.2022.11.006 36403366

[105] Aronson SL Lopez-Yurda M Koole SN Schagen van Leeuwen JH Schreuder HWR Hermans RHM. Cytoreductive surgery with or without hyperthermic intraperitoneal chemotherapy in patients with advanced ovarian cancer (OVHIPEC-1): final survival analysis of a randomised, controlled, phase 3 trial. Lancet Oncol. (2023) 24(10):1109–18. doi: 10.1016/S1470-2045(23)00396-0 37708912

[106] Schouten PC Richters L Vis DJ Kommoss S van Dijk E Ernst C. Ovarian Cancer-Specific BRCA-like Copy-Number Aberration Classifiers Detect Mutations Associated with Homologous Recombination Deficiency in the AGO-TR1 Trial. Clin Cancer Res. (2021) 27(23):6559–69. doi: 10.1158/1078-0432.Ccr-21-1673 PMC9401539 34593530

[107] Ghirardi V Trozzi R Scambia G Fagotti A. Current and future trials about HIPEC in ovarian cancer. Bull du Cancer. (2024) 111(3):254–60. doi: 10.1016/j.bulcan.2023.01.016 36863924

[108] Goel N Foxall ME Scalise CB Wall JA Arend RC. Strategies in Overcoming Homologous Recombination Proficiency and PARP Inhibitor Resistance. Mol Cancer Ther. (2021) 20(9):1542–9. doi: 10.1158/1535-7163.Mct-20-0992 PMC9066363 34172532

[109] Pascal JM. The comings and goings of PARP-1 in response to DNA damage. DNA Repair (Amst). (2018) 71:177–82. doi: 10.1016/j.dnarep.2018.08.022 PMC6637744 30177435

[110] Mirza MR Monk BJ Herrstedt J Oza AM Mahner S Redondo A. Niraparib Maintenance Therapy in Platinum-Sensitive, Recurrent Ovarian Cancer. N Engl J Med. (2016) 375(22):2154–64. doi: 10.1056/NEJMoa1611310 27717299

[111] del Campo JM Matulonis UA Malander S Provencher D Mahner S Follana P. Niraparib Maintenance Therapy in Patients With Recurrent Ovarian Cancer After a Partial Response to the Last Platinum-Based Chemotherapy in the ENGOT-OV16/NOVA Trial. J Clin Oncol. (2019) 37(32):2968–73. doi: 10.1200/JCO.18.02238 PMC6839909 31173551

[112] You B Sehgal V Hosmane B Huang X Ansell PJ Dinh MH. CA-125 KELIM as a Potential Complementary Tool for Predicting Veliparib Benefit: An Exploratory Analysis From the VELIA/GOG-3005 Study. J Clin Oncol. (2023) 41(1):107–16. doi: 10.1200/jco.22.00430 PMC9788978 35867965

[113] Swisher EM Aghajanian C O'Malley DM Fleming GF Kaufmann SH Levine DA. Impact of homologous recombination status and responses with veliparib combined with first-line chemotherapy in ovarian cancer in the Phase 3 VELIA/GOG-3005 study. Gynecologic Oncol. (2022) 164(2):245–53. 10.1016/j.ygyno.2021.12.003 34906376

[114] Shibuya M. Vascular Endothelial Growth Factor (VEGF) and Its Receptor (VEGFR) Signaling in Angiogenesis: A Crucial Target for Anti- and Pro-Angiogenic Therapies. Genes Cancer. (2011) 2(12):1097–105. doi: 10.1177/1947601911423031 PMC3411125 22866201

[115] Tewari KS Burger RA Enserro D Norquist BM Swisher EM Brady MF. Final Overall Survival of a Randomized Trial of Bevacizumab for Primary Treatment of Ovarian Cancer. J Clin Oncol. (2019) 37(26):2317–28. doi: 10.1200/jco.19.01009 PMC6879307 31216226

[116] Nakai H Matsumura N. The roles and limitations of bevacizumab in the treatment of ovarian cancer. Int J Clin Oncol. (2022) 27(7):1120–6. doi: 10.1007/s10147-022-02169-x 35477830

[117] Chan N Bristow RG. "Contextual" synthetic lethality and/or loss of heterozygosity: tumor hypoxia and modification of DNA repair. Clin Cancer Res. (2010) 16(18):4553–60. doi: 10.1158/1078-0432.Ccr-10-0527 20823145

[118] Ribeiro ARG Salvadori MM de Brot L Bovolin G Mantoan H Ilelis F. Retrospective analysis of the role of cyclin E1 overexpression as a predictive marker for the efficacy of bevacizumab in platinum-sensitive recurrent ovarian cancer. Ecancermedicalscience. (2021) 15:1262. doi: 10.3332/ecancer.2021.1262 34567247 PMC8426016

[119] Mirza MR Åvall Lundqvist E Birrer MJ dePont Christensen R Nyvang GB Malander S. Niraparib plus bevacizumab versus niraparib alone for platinum-sensitive recurrent ovarian cancer (NSGO-AVANOVA2/ENGOT-ov24): a randomised, phase 2, superiority trial. Lancet Oncol. (2019) 20(10):1409–19. doi: 10.1016/s1470-2045(19)30515-7 31474354

[120] Norquist BM Brady MF Harrell MI Walsh T Lee MK Gulsuner S. Mutations in Homologous Recombination Genes and Outcomes in Ovarian Carcinoma Patients in GOG 218: An NRG Oncology/Gynecologic Oncology Group Study. Clin Cancer Res. (2018) 24(4):777–83. doi: 10.1158/1078-0432.Ccr-17-1327 PMC5815909 29191972

[121] Teresa Z Intidhar L-G Maria Del G Cristiana S Ilaria C. The clinical challenges of homologous recombination proficiency in ovarian cancer: from intrinsic resistance to new treatment opportunities. Cancer Drug Resistance. (2023) 6(3):499–516. doi: 10.20517/cdr.2023.08 37842243 PMC10571062

[122] Lim JJ Yang K Taylor-Harding B Wiedemeyer WR Buckanovich RJ. VEGFR3 inhibition chemosensitizes ovarian cancer stemlike cells through down-regulation of BRCA1 and BRCA2. Neoplasia. (2014) 16(4):343–53. doi: 10.1016/j.neo.2014.04.003 PMC4094836 24862760

[123] Liu JF Barry WT Birrer M Lee JM Buckanovich RJ Fleming GF. Combination cediranib and olaparib versus olaparib alone for women with recurrent platinum-sensitive ovarian cancer: a randomised phase 2 study. Lancet Oncol. (2014) 15(11):1207–14. doi: 10.1016/s1470-2045(14)70391-2 PMC4294183 25218906

[124] Bizzaro F Fuso Nerini I Taylor MA Anastasia A Russo M Damia G. VEGF pathway inhibition potentiates PARP inhibitor efficacy in ovarian cancer independent of BRCA status. J Hematol Oncol. (2021) 14(1):186. doi: 10.1186/s13045-021-01196-x 34742344 PMC8572452

[125] Lee J-M Moore RG Ghamande S Park MS Diaz JP Chapman J. Cediranib in Combination with Olaparib in Patients without a Germline BRCA1/2 Mutation and with Recurrent Platinum-Resistant Ovarian Cancer: Phase IIb CONCERTO Trial. Clin Cancer Res. (2022) 28(19):4186–93. doi: 10.1158/1078-0432.Ccr-21-1733 PMC9527502 35917514

[126] Liu JF Brady MF Matulonis UA Miller A Kohn EC Swisher EM. Olaparib With or Without Cediranib Versus Platinum-Based Chemotherapy in Recurrent Platinum-Sensitive Ovarian Cancer (NRG-GY004): A Randomized, Open-Label, Phase III Trial. J Clin Oncol. (2022) 40(19):2138–47. doi: 10.1200/jco.21.02011 PMC9242406 35290101

[127] Cunnea P Curry EW Christie EL Nixon K Kwok CH Pandey A. Spatial and temporal intra-tumoral heterogeneity in advanced HGSOC: Implications for surgical and clinical outcomes. Cell Rep Med. (2023) 4(6):101055. doi: 10.1016/j.xcrm.2023.101055 37220750 PMC10313917

[128] Hall M Savvatis K Nixon K Kyrgiou M Hariharan K Padwick M. Maximal-Effort Cytoreductive Surgery for Ovarian Cancer Patients with a High Tumor Burden: Variations in Practice and Impact on Outcome. Annals of Surgical Oncology. (2019) 26(9):2943–51. doi: 10.1245/s10434-019-07516-3 PMC6682567 31243666

[129] Shi T Zhu J Feng Y Tu D Zhang Y Zhang P. Secondary cytoreduction followed by chemotherapy versus chemotherapy alone in platinum-sensitive relapsed ovarian cancer (SOC-1): a multicentre, open-label, randomised, phase 3 trial. Lancet Oncol. (2021) 22(4):439–49. doi: 10.1016/s1470-2045(21)00006-1 33705695

[130] Harter P Sehouli J Vergote I Ferron G Reuss A Meier W. Randomized Trial of Cytoreductive Surgery for Relapsed Ovarian Cancer. N Engl J Med. (2021) 385(23):2123–31. doi: 10.1056/NEJMoa2103294 34874631

[131] Coleman RL Spirtos NM Enserro D Herzog TJ Sabbatini P Armstrong DK. Secondary Surgical Cytoreduction for Recurrent Ovarian Cancer. New Engl J Med. (2019) 381(20):1929–39. doi: 10.1056/NEJMoa1902626 PMC6941470 31722153

[132] Petrillo M Sozzi G Dessole M Capobianco G Dessole S Madonia M. The role of surgery in platinum-resistant ovarian cancer: A call to the scientific community. Semin Cancer Biol. (2021) 77:194–202. doi: 10.1016/j.semcancer.2021.02.009 33607247

[133] Zwimpfer TA Tal O Geissler F Coelho R Rimmer N Jacob F. Low grade serous ovarian cancer - A rare disease with increasing therapeutic options. Cancer Treat Rev. (2023) 112:102497. doi: 10.1016/j.ctrv.2022.102497 36525716

[134] Petrillo M Pedone Anchora L Tortorella L Fanfani F Gallotta V Pacciani M. Secondary cytoreductive surgery in patients with isolated platinum-resistant recurrent ovarian cancer: A retrospective analysis. Gynecologic Oncol. (2014) 134(2):257–61. doi: 10.1016/j.ygyno.2014.05.029 24910451

[135] Musella A Marchetti C Palaia I Perniola G Giorgini M Lecce F. Secondary Cytoreduction in Platinum-Resistant Recurrent Ovarian Cancer: A Single-Institution Experience. Ann Surg Oncol. (2015) 22(13):4211–6. doi: 10.1245/s10434-015-4523-2 25801357

[136] Tuninetti V Di Napoli M Ghisoni E Maggiorotto F Robella M Scotto G. Cytoreductive Surgery for Heavily Pre-Treated, Platinum-Resistant Epithelial Ovarian Carcinoma: A Two-Center Retrospective Experience. Cancers. (2020) 12(8):2239. 32785193 10.3390/cancers12082239 PMC7464658

[137] Marchetti C De Leo R Musella A D'Indinosante M Capoluongo E Minucci A. BRCA Mutation Status to Personalize Management of Recurrent Ovarian Cancer: A Multicenter Study. Ann Surg Oncol. (2018) 25(12):3701–8. doi: 10.1245/s10434-018-6700-6 30128899

[138] Fan CA Reader J Roque DM. Review of Immune Therapies Targeting Ovarian Cancer. Curr Treat Options Oncol. (2018) 19(12):74. doi: 10.1007/s11864-018-0584-3 30430276

[139] Colombo I Karakasis K Suku S Oza AM. Chasing Immune Checkpoint Inhibitors in Ovarian Cancer: Novel Combinations and Biomarker Discovery. Cancers (Basel). (2023) 15(12):3220. doi: 10.3390/cancers15123220 37370830 PMC10296292

[140] Lee EK Xiong N Cheng SC Barry WT Penson RT Konstantinopoulos PA. Combined pembrolizumab and pegylated liposomal doxorubicin in platinum resistant ovarian cancer: A phase 2 clinical trial. Gynecol Oncol. (2020) 159(1):72–8. doi: 10.1016/j.ygyno.2020.07.028 32771276

[141] Pujade-Lauraine E Fujiwara K Ledermann JA Oza AM Kristeleit R Ray-Coquard IL. Avelumab alone or in combination with chemotherapy versus chemotherapy alone in platinum-resistant or platinum-refractory ovarian cancer (JAVELIN Ovarian 200): an open-label, three-arm, randomised, phase 3 study. Lancet Oncol. (2021) 22(7):1034–46. doi: 10.1016/s1470-2045(21)00216-3 34143970

[142] Monk BJ Colombo N Oza AM Fujiwara K Birrer MJ Randall L. Chemotherapy with or without avelumab followed by avelumab maintenance versus chemotherapy alone in patients with previously untreated epithelial ovarian cancer (JAVELIN Ovarian 100): an open-label, randomised, phase 3 trial. Lancet Oncol. (2021) 22(9):1275–89. doi: 10.1016/s1470-2045(21)00342-9 34363762

[143] Riccardi F Dal Bo M Macor P Toffoli G. A comprehensive overview on antibody-drug conjugates: from the conceptualization to cancer therapy. Front Pharmacol. (2023) 14:1274088. doi: 10.3389/fphar.2023.1274088 37790810 PMC10544916

[144] Moore KN Angelergues A Konecny GE García Y Banerjee S Lorusso D. Mirvetuximab Soravtansine in FRα-Positive, Platinum-Resistant Ovarian Cancer. New Engl J Med. (2023) 389(23):2162–74. doi: 10.1056/NEJMoa2309169 38055253

[145] Kalli KR Oberg AL Keeney GL Christianson TJ Low PS Knutson KL. Folate receptor alpha as a tumor target in epithelial ovarian cancer. Gynecol Oncol. (2008) 108(3):619–26. doi: 10.1016/j.ygyno.2007.11.020 PMC2707764 18222534

[146] Morand S Devanaboyina M Staats H Stanbery L Nemunaitis J. Ovarian Cancer Immunotherapy and Personalized Medicine. Int J Mol Sci. (2021) 22(12):6532. doi: 10.3390/ijms22126532 34207103 PMC8234871

[147] Oh J Barve M Matthews CM Koon EC Heffernan TP Fine B. Phase II study of Vigil® DNA engineered immunotherapy as maintenance in advanced stage ovarian cancer. Gynecologic Oncol. (2016) 143(3):504–10. doi: 10.1016/j.ygyno.2016.09.018 27678295

[148] Bristow RE Baldwin RL Yamada SD Korc M Karlan BY. Altered expression of transforming growth factor-β ligands and receptors in primary and recurrent ovarian carcinoma. Cancer. (1999) 85(3):658–68. doi: 10.1002/(SICI)1097-0142(19990201)85:3<658::AID-CNCR16>3.0.CO;2-M 10091739

[149] Zhang Y Zhang L Zhao Y Wang S Feng L. Efficacy and safety of Gemogenovatucel-T (Vigil) immunotherapy for advanced ovarian carcinoma: A systematic review and meta-analysis of randomized controlled trials. Front Oncol. (2022) 12:945867. doi: 10.3389/fonc.2022.945867 36338747 PMC9634109

[150] Senzer N Barve M Nemunaitis J Kuhn J Melnyk A Beitsch P. Long term follow up: phase I trial of “bi-shRNA furin/GMCSF DNA/autologous tumor cell” immunotherapy (FANG™) in advanced cancer. J Vaccines Vaccin. (2013) 4(8):209. doi: 10.4172/2157-7560.1000209

[151] Rocconi RP Grosen EA Ghamande SA Chan JK Barve MA Oh J. Gemogenovatucel-T (Vigil) immunotherapy as maintenance in frontline stage III/IV ovarian cancer (VITAL): a randomised, double-blind, placebo-controlled, phase 2b trial. Lancet Oncol. (2020) 21(12):1661–72. doi: 10.1016/S1470-2045(20)30533-7 33271095

[152] Rocconi RP Monk BJ Walter A Herzog TJ Galanis E Manning L. Gemogenovatucel-T (Vigil) immunotherapy demonstrates clinical benefit in homologous recombination proficient (HRP) ovarian cancer. Gynecol Oncol. (2021) 161(3):676–80. doi: 10.1016/j.ygyno.2021.03.009 33715892

[153] McGranahan N Furness AJS Rosenthal R Ramskov S Lyngaa R Saini SK. Clonal neoantigens elicit T cell immunoreactivity and sensitivity to immune checkpoint blockade. Science. (2016) 351(6280):1463–9. doi: 10.1126/science.aaf1490 PMC4984254 26940869

[154] Preston CC Goode EL Hartmann LC Kalli KR Knutson KL. Immunity and immune suppression in human ovarian cancer. Immunotherapy. (2011) 3(4):539–56. doi: 10.2217/imt.11.20 PMC3147144 21463194

[155] Rocconi RP Stevens EE Bottsford-Miller JN Ghamande SA Elder J DeMars LL. Proof of principle study of sequential combination atezolizumab and Vigil in relapsed ovarian cancer. Cancer Gene Ther. (2022) 29(3-4):369–82. doi: 10.1038/s41417-021-00317-5 33753870

[156] Sarivalasis A Morotti M Mulvey A Imbimbo M Coukos G. Cell therapies in ovarian cancer. Ther Adv Med Oncol. (2021) 13:17588359211008399. doi: 10.1177/17588359211008399 33995591 PMC8072818

[157] Drew Y Kim JW Penson RT O'Malley DM Parkinson C Roxburgh P. Olaparib plus Durvalumab, with or without Bevacizumab, as Treatment in PARP Inhibitor-Naïve Platinum-Sensitive Relapsed Ovarian Cancer: A Phase II Multi-Cohort Study. Clin Cancer Res. (2024) 30(1):50–62. doi: 10.1158/1078-0432.Ccr-23-2249 37939124 PMC10767301

[158] Harter P Trillsch F Okamoto A Reuss A Kim J-W Rubio-Pérez MJ. Durvalumab with paclitaxel/carboplatin (PC) and bevacizumab (bev), followed by maintenance durvalumab, bev, and olaparib in patients (pts) with newly diagnosed advanced ovarian cancer (AOC) without a tumor BRCA1/2 mutation (non-tBRCAm): Results from the randomized, placebo (pbo)-controlled phase III DUO-O trial. J Clin Oncol. (2023) 41(17_suppl):LBA5506–LBA. doi: 10.1200/JCO.2023.41.17_suppl.LBA5506

[159] Shapiro G Do K Tolaney S Hilton J Cleary J Wolanski A. Abstract CT047: Phase 1 dose-escalation study of the CDK inhibitor dinaciclib in combination with the PARP inhibitor veliparib in patients with advanced solid tumors. Cancer Res. (2017) 77:CT047–CT. doi: 10.1158/1538-7445.AM2017-CT047

[160] Westin SN Coleman RL Fellman BM Yuan Y Sood AK Soliman PT. EFFORT: EFFicacy Of adavosertib in parp ResisTance: A randomized two-arm non-comparative phase II study of adavosertib with or without olaparib in women with PARP-resistant ovarian cancer. J Clin Oncol. (2021) 39(15_suppl):5505. doi: 10.1200/JCO.2021.39.15_suppl.5505

[161] Konstantinopoulos PA Gonzalez-Martin A Cruz FM Friedlander M Glasspool R Lorusso D. EPIK-O/ENGOT-OV61: alpelisib plus olaparib vs cytotoxic chemotherapy in high-grade serous ovarian cancer (phase III study). Future Oncol. (2022) 18(31):3481–92. doi: 10.2217/fon-2022-0666 36066851

[162] Konstantinopoulos PA Cheng SC Supko JG Polak M Wahner-Hendrickson AE Ivy SP. Combined PARP and HSP90 inhibition: preclinical and Phase 1 evaluation in patients with advanced solid tumours. Br J Cancer. (2022) 126(7):1027–36. doi: 10.1038/s41416-021-01664-8 PMC8980096 34887522

[163] Fang D Huang S Su SB. Cyclin E1-CDK 2, a potential anticancer target. Aging (Albany NY). (2016) 8(4):571–2. doi: 10.18632/aging.100946 PMC4925813 27085092

[164] Chen XX Xie FF Zhu XJ Lin F Pan SS Gong LH. Cyclin-dependent kinase inhibitor dinaciclib potently synergizes with cisplatin in preclinical models of ovarian cancer. Oncotarget. (2015) 6(17):14926–39. doi: 10.18632/oncotarget.3717 PMC4558126 25962959

[165] Johnson N Li YC Walton ZE Cheng KA Li D Rodig SJ. Compromised CDK1 activity sensitizes BRCA-proficient cancers to PARP inhibition. Nat Med. (2011) 17(7):875–82. doi: 10.1038/nm.2377 PMC3272302 21706030

[166] Johnson SF Johnson N Chi D Primack B D'Andrea AD Lim E. Abstract 1788: The CDK inhibitor dinaciclib sensitizes triple-negative breast cancer cells to PARP inhibition. Cancer Res. 2013 73(8_Supplement):1788–. doi: 10.1158/1538-7445.Am2013-1788

[167] Chen P Lee NV Hu W Xu M Ferre RA Lam H. Spectrum and Degree of CDK Drug Interactions Predicts Clinical Performance. Mol Cancer Ther. (2016) 15(10):2273–81. doi: 10.1158/1535-7163.Mct-16-0300 27496135

[168] Au-Yeung G Bressel M Prall O Surace D Andrews J Mongta S. IGNITE: A phase II signal-seeking trial of adavosertib targeting recurrent high-grade, serous ovarian cancer with cyclin E1 overexpression with and without gene amplification. J Clin Oncol. (2022) 40(16_suppl):5515–. doi: 10.1200/JCO.2022.40.16_suppl.5515

[169] Johnson N Cai D Kennedy RD Pathania S Arora M Li YC. Cdk1 participates in BRCA1-dependent S phase checkpoint control in response to DNA damage. Mol Cell. (2009) 35(3):327–39. doi: 10.1016/j.molcel.2009.06.036 PMC3024055 19683496

[170] Turner N Tutt A Ashworth A. Hallmarks of 'BRCAness' in sporadic cancers. Nat Rev Cancer. (2004) 4(10):814–9. doi: 10.1038/nrc1457 15510162

[171] Kausar T Schreiber JS Karnak D Parsels LA Parsels JD Davis MA. Sensitization of Pancreatic Cancers to Gemcitabine Chemoradiation by WEE1 Kinase Inhibition Depends on Homologous Recombination Repair. Neoplasia. (2015) 17(10):757–66. doi: 10.1016/j.neo.2015.09.006 PMC4656803 26585231

[172] Parsels LA Karnak D Parsels JD Zhang Q Vélez-Padilla J Reichert ZR. PARP1 Trapping and DNA Replication Stress Enhance Radiosensitization with Combined WEE1 and PARP Inhibitors. Mol Cancer Res. (2018) 16(2):222–32. doi: 10.1158/1541-7786.Mcr-17-0455 PMC5805596 29133592

[173] Schutte T Embaby A Steeghs N van der Mierden S van Driel W Rijlaarsdam M. Clinical development of WEE1 inhibitors in gynecological cancers: A systematic review. Cancer Treat Rev. (2023) 115:102531. doi: 10.1016/j.ctrv.2023.102531 36893690

[174] Zhou S Yung Chan S Cher Goh B Chan E Duan W Huang M. Mechanism-based inhibition of cytochrome P450 3A4 by therapeutic drugs. Clin Pharmacokinet. (2005) 44(3):279–304. doi: 10.2165/00003088-200544030-00005 15762770

[175] Heijink AM Blomen VA Bisteau X Degener F Matsushita FY Kaldis P. A haploid genetic screen identifies the G1/S regulatory machinery as a determinant of Wee1 inhibitor sensitivity. Proc Natl Acad Sci U.S.A. (2015) 112(49):15160–5. doi: 10.1073/pnas.1505283112 PMC4679052 26598692

[176] Domínguez-Kelly R Martín Y Koundrioukoff S Tanenbaum ME Smits VA Medema RH. Wee1 controls genomic stability during replication by regulating the Mus81-Eme1 endonuclease. J Cell Biol. (2011) 194(4):567–79. doi: 10.1083/jcb.201101047 PMC3160579 21859861

[177] Fruman DA Chiu H Hopkins BD Bagrodia S Cantley LC Abraham RT. The PI3K Pathway in Human Disease. Cell. (2017) 170(4):605–35. doi: 10.1016/j.cell.2017.07.029 PMC5726441 28802037

[178] LoRusso PM. Inhibition of the PI3K/AKT/mTOR Pathway in Solid Tumors. J Clin Oncol. (2016) 34(31):3803–15. doi: 10.1200/jco.2014.59.0018 PMC6366304 27621407

[179] Ibrahim YH García-García C Serra V He L Torres-Lockhart K Prat A. PI3K inhibition impairs BRCA1/2 expression and sensitizes BRCA-proficient triple-negative breast cancer to PARP inhibition. Cancer Discovery. (2012) 2(11):1036–47. doi: 10.1158/2159-8290.Cd-11-0348 PMC5125254 22915752

[180] Liu JF Palakurthi S Zeng Q Zhou S Ivanova E Huang W. Establishment of Patient-Derived Tumor Xenograft Models of Epithelial Ovarian Cancer for Preclinical Evaluation of Novel Therapeutics. Clin Cancer Res. (2017) 23(5):1263–73. doi: 10.1158/1078-0432.Ccr-16-1237 PMC5332350 27573169

[181] Konstantinopoulos PA Barry WT Birrer M Westin SN Cadoo KA Shapiro GI. Olaparib and α-specific PI3K inhibitor alpelisib for patients with epithelial ovarian cancer: a dose-escalation and dose-expansion phase 1b trial. Lancet Oncol. (2019) 20(4):570–80. doi: 10.1016/s1470-2045(18)30905-7 PMC7025391 30880072

[182] Rascio F Spadaccino F Rocchetti MT Castellano G Stallone G Netti GS. The Pathogenic Role of PI3K/AKT Pathway in Cancer Onset and Drug Resistance: An Updated Review. Cancers (Basel). (2021) 13(16):3949. doi: 10.3390/cancers13163949 34439105 PMC8394096

[183] Westin SN Labrie M Litton JK Blucher A Fang Y Vellano CP. Phase Ib Dose Expansion and Translational Analyses of Olaparib in Combination with Capivasertib in Recurrent Endometrial, Triple-Negative Breast, and Ovarian Cancer. Clin Cancer Res. (2021) 27(23):6354–65. doi: 10.1158/1078-0432.Ccr-21-1656 PMC8639651 34518313

[184] Turner NC Oliveira M Howell SJ Dalenc F Cortes J Gomez Moreno HL. Capivasertib in Hormone Receptor–Positive Advanced Breast Cancer. New Engl J Med. (2023) 388(22):2058–70. doi: 10.1056/NEJMoa2214131 PMC11335038 37256976

[185] Kim H Xu H George E Hallberg D Kumar S Jagannathan V. Combining PARP with ATR inhibition overcomes PARP inhibitor and platinum resistance in ovarian cancer models. Nat Commun. (2020) 11(1):3726. doi: 10.1038/s41467-020-17127-2 32709856 PMC7381609

[186] Wethington SL Shah PD Martin LP Tanyi JL Latif NA Morgan MA. Combination of PARP and ATR inhibitors (olaparib and ceralasertib) shows clinical activity in acquired PARP inhibitor-resistant recurrent ovarian cancer. J Clin Oncol. (2021) 39(15_suppl):5516–. doi: 10.1200/JCO.2021.39.15_suppl.5516

[187] Wethington SL Shah PD Martin L Tanyi JL Latif N Morgan M. Combination ATR (ceralasertib) and PARP (olaparib) Inhibitor (CAPRI) Trial in Acquired PARP Inhibitor-Resistant Homologous Recombination-Deficient Ovarian Cancer. Clin Cancer Res. (2023) 29(15):2800–7. doi: 10.1158/1078-0432.Ccr-22-2444 PMC11934101 37097611

[188] Yap TA Fontana E Lee EK Spigel DR Højgaard M Lheureux S. Camonsertib in DNA damage response-deficient advanced solid tumors: phase 1 trial results. Nat Med. (2023) 29(6):1400–11. doi: 10.1038/s41591-023-02399-0 PMC10287555 37277454

[189] Yap TA Tan DSP Terbuch A Caldwell R Guo C Goh BC. First-in-Human Trial of the Oral Ataxia Telangiectasia and RAD3-Related (ATR) Inhibitor BAY 1895344 in Patients with Advanced Solid Tumors. Cancer Discovery. (2021) 11(1):80–91. doi: 10.1158/2159-8290.Cd-20-0868 32988960 PMC9554790

[190] Bradbury A Zenke FT Curtin NJ Drew Y. The Role of ATR Inhibitors in Ovarian Cancer: Investigating Predictive Biomarkers of Response. Cells. (2022) 11(15):2361. doi: 10.3390/cells11152361 35954206 PMC9367423

[191] Lee JM Nair J Zimmer A Lipkowitz S Annunziata CM Merino MJ. Prexasertib, a cell cycle checkpoint kinase 1 and 2 inhibitor, in BRCA wild-type recurrent high-grade serous ovarian cancer: a first-in-class proof-of-concept phase 2 study. Lancet Oncol. (2018) 19(2):207–15. doi: 10.1016/s1470-2045(18)30009-3 PMC7366122 29361470

[192] Gupta N Huang TT Nair JR An D Zurcher G Lampert EJ. BLM overexpression as a predictive biomarker for CHK1 inhibitor response in PARP inhibitor-resistant BRCA-mutant ovarian cancer. Sci Transl Med. (2023) 15(701):eadd7872. doi: 10.1126/scitranslmed.add7872 37343085 PMC10758289

[193] Giudice E Huang TT Nair JR Zurcher G McCoy A Nousome D. The CHK1 inhibitor prexasertib in BRCA wild-type platinum-resistant recurrent high-grade serous ovarian carcinoma: a phase 2 trial. Nat Commun. (2024) 15(1):2805. doi: 10.1038/s41467-024-47215-6 38555285 PMC10981752

[194] Kim MG Pak JH Choi WH Park JY Nam JH Kim JH. The relationship between cisplatin resistance and histone deacetylase isoform overexpression in epithelial ovarian cancer cell lines. J Gynecol Oncol. (2012) 23(3):182–9. doi: 10.3802/jgo.2012.23.3.182 PMC3395014 22808361

[195] Koprinarova M Botev P Russev G. Histone deacetylase inhibitor sodium butyrate enhances cellular radiosensitivity by inhibiting both DNA nonhomologous end joining and homologous recombination. DNA Repair (Amst). (2011) 10(9):970–7. doi: 10.1016/j.dnarep.2011.07.003 21824827

[196] Adimoolam S Sirisawad M Chen J Thiemann P Ford JM Buggy JJ. HDAC inhibitor PCI-24781 decreases RAD51 expression and inhibits homologous recombination. Proc Natl Acad Sci U S A. (2007) 104(49):19482–7. doi: 10.1073/pnas.0707828104 PMC2148315 18042714

[197] Konstantinopoulos PA Wilson AJ Saskowski J Wass E Khabele D. Suberoylanilide hydroxamic acid (SAHA) enhances olaparib activity by targeting homologous recombination DNA repair in ovarian cancer. Gynecol Oncol. (2014) 133(3):599–606. doi: 10.1016/j.ygyno.2014.03.007 24631446 PMC4347923

[198] Wilson AJ Sarfo-Kantanka K Barrack T Steck A Saskowski J Crispens MA. Panobinostat sensitizes cyclin E high, homologous recombination-proficient ovarian cancer to olaparib. Gynecol Oncol. (2016) 143(1):143–51. doi: 10.1016/j.ygyno.2016.07.088 PMC5031537 27444036

[199] Wilson AJ Lalani AS Wass E Saskowski J Khabele D. Romidepsin (FK228) combined with cisplatin stimulates DNA damage-induced cell death in ovarian cancer. Gynecol Oncol. (2012) 127(3):579–86. doi: 10.1016/j.ygyno.2012.09.016 PMC3541411 23010348

[200] Gupta VG Hirst J Petersen S Roby KF Kusch M Zhou H. Entinostat, a selective HDAC1/2 inhibitor, potentiates the effects of olaparib in homologous recombination proficient ovarian cancer. Gynecol Oncol. (2021) 162(1):163–72. doi: 10.1016/j.ygyno.2021.04.015 PMC8647995 33867143

[201] Gabbasov R Benrubi ID O'Brien SW Krais JJ Johnson N Litwin S. Targeted blockade of HSP90 impairs DNA-damage response proteins and increases the sensitivity of ovarian carcinoma cells to PARP inhibition. Cancer Biol Ther. (2019) 20(7):1035–45. doi: 10.1080/15384047.2019.1595279 PMC6606007 30929564

[202] Choi YE Battelli C Watson J Liu J Curtis J Morse AN. Sublethal concentrations of 17-AAG suppress homologous recombination DNA repair and enhance sensitivity to carboplatin and olaparib in HR proficient ovarian cancer cells. Oncotarget. (2014) 5(9):2678–87. doi: 10.18632/oncotarget.1929 PMC4058036 24798692

[203] Hoy SM. Pimitespib: First Approval. Drugs. (2022) 82(13):1413–8. doi: 10.1007/s40265-022-01764-6 35986838

[204] Donati B Lorenzini E Ciarrocchi A. BRD4 and Cancer: going beyond transcriptional regulation. Mol Cancer. (2018) 17(1):164. doi: 10.1186/s12943-018-0915-9 30466442 PMC6251205

[205] Baratta MG Schinzel AC Zwang Y Bandopadhayay P Bowman-Colin C Kutt J. An in-tumor genetic screen reveals that the BET bromodomain protein, BRD4, is a potential therapeutic target in ovarian carcinoma. Proc Natl Acad Sci U.S.A. (2015) 112(1):232–7. doi: 10.1073/pnas.1422165112 PMC4291641 25535366

[206] Rhyasen GW Yao Y Zhang J Dulak A Castriotta L Jacques K. BRD4 amplification facilitates an oncogenic gene expression program in high-grade serous ovarian cancer and confers sensitivity to BET inhibitors. PloS One. (2018) 13(7):e0200826. doi: 10.1371/journal.pone.0200826 30036377 PMC6056044

[207] Drumond-Bock AL Bieniasz M. The role of distinct BRD4 isoforms and their contribution to high-grade serous ovarian carcinoma pathogenesis. Mol Cancer. (2021) 20(1):145. doi: 10.1186/s12943-021-01424-5 34758842 PMC8579545

[208] Pan-cancer analysis of whole genomes. Nature. (2020) 578(7793):82–93. doi: 10.1038/s41586-020-1969-6 32025007 PMC7025898

[209] Andrikopoulou A Liontos M Koutsoukos K Dimopoulos MA Zagouri F. Clinical perspectives of BET inhibition in ovarian cancer. Cell Oncol (Dordr). (2021) 44(2):237–49. doi: 10.1007/s13402-020-00578-6 PMC12980713 33469840

[210] Karakashev S Zhu H Yokoyama Y Zhao B Fatkhutdinov N Kossenkov AV. BET Bromodomain Inhibition Synergizes with PARP Inhibitor in Epithelial Ovarian Cancer. Cell Rep. (2017) 21(12):3398–405. doi: 10.1016/j.celrep.2017.11.095 PMC5745042 29262321

[211] Watanabe N Broome M Hunter T. Regulation of the human WEE1Hu CDK tyrosine 15-kinase during the cell cycle. EMBO J. (1995) 14(9):1878–91. doi: 10.1002/j.1460-2075.1995.tb07180.x PMC398287 7743995

[212] Mir SE De Witt Hamer PC Krawczyk PM Balaj L Claes A Niers JM. In silico analysis of kinase expression identifies WEE1 as a gatekeeper against mitotic catastrophe in glioblastoma. Cancer Cell. (2010) 18(3):244–57. doi: 10.1016/j.ccr.2010.08.011 PMC3115571 20832752

[213] Yan S Michael WM. TopBP1 and DNA polymerase alpha-mediated recruitment of the 9-1-1 complex to stalled replication forks: implications for a replication restart-based mechanism for ATR checkpoint activation. Cell Cycle. (2009) 8(18):2877–84. doi: 10.4161/cc.8.18.9485 19652550

[214] Wilson AJ Stubbs M Liu P Ruggeri B Khabele D. The BET inhibitor INCB054329 reduces homologous recombination efficiency and augments PARP inhibitor activity in ovarian cancer. Gynecol Oncol. (2018) 149(3):575–84. doi: 10.1016/j.ygyno.2018.03.049 PMC5986599 29567272

[215] Bechter O Schöffski P. Make your best BET: The emerging role of BET inhibitor treatment in malignant tumors. Pharmacol Ther. (2020) 208:107479. doi: 10.1016/j.pharmthera.2020.107479 31931101

[216] Bock C Datlinger P Chardon F Coelho MA Dong MB Lawson KA. High-content CRISPR screening. Nat Rev Methods Primers. (2022) 2(1):9. doi: 10.1038/s43586-022-00098-7 37214176 PMC10200264

[217] Zhang J Li Y Liu H Zhang J Wang J Xia J. Genome-wide CRISPR/Cas9 library screen identifies PCMT1 as a critical driver of ovarian cancer metastasis. J Exp Clin Cancer Res. (2022) 41(1):24. doi: 10.1186/s13046-022-02242-3 35033172 PMC8760697

