# Supplementary material for: Homologous recombination proficient subtypes of high-grade serous ovarian cancer: treatment options for a poor prognosis group
Source: Front Oncol. 2024 Jun 4;14:1387281. doi: 10.3389/fonc.2024.1387281 (PMC11183307; doi:10.3389/fonc.2024.1387281)
Supplement: Supplementary file 1 [file DataSheet_1.docx]

**Supplementary material**: Overview of the search terms that were used.

| **Topic** | **Search term** | **Combinations** |
| --- | --- | --- |
| HGSC | Homologous recombination repair OR HRR  Homologous recombination proficiency OR HRP  Non-HRD  HR status  (High grade serous cancer OR tubo ovarian high grade serous carcinoma OR HGSC) pathogenesis  CCNE1 amplification  AKT2 amplification  CDK1/2 alteration  HRD status  Mutation status  Genomic instability assays  Functional assays  Recurrence  Firstline treatment  Maintenance treatment  ESGO-ESMO-ESP | (Homologous recombination proficiency OR HRP) vs (Homologous recombination deficiency OR HRD)  (Homologous recombination proficiency OR HRP) AND characteristics  (Homologous recombination proficiency OR HRP) AND molecular  (Homologous recombination proficiency OR HRP) AND age  (Homologous recombination proficiency OR HRP) AND population  (Homologous recombination proficiency OR HRP) AND disease  (Homologous recombination proficiency OR HRP) AND (Progression free survival OR PFS)  (Homologous recombination proficiency OR HRP) AND (Overall survival OR OS)  (Homologous recombination proficiency OR HRP) AND outcome  (Homologous recombination proficiency OR HRP) AND immune profile  (Homologous recombination proficiency OR HRP) AND subtypes  (Homologous recombination proficiency OR HRP) AND biomarker  (Homologous recombination proficiency OR HRP) AND stratification  (Homologous recombination proficiency OR HRP) AND treatment |
| Pathways | Homologous recombination pathway  HR genes  BRCAness  CDK  Pl3K/AKT/mTOR  DNA replication stress | (Homologous recombination repair OR HRR) AND pathway inactivation  Cyclin E1/CDK2 complex  BRCA AND phosphorylation |
| Treatment resistance | Primary platinum resistance  Reversion of HR gene  Acquired (Homologous recombination  proficiency OR HRP)  Recurrent ovarian cancer | (Homologous recombination proficiency OR HRP) AND treatment resistance |
| Cytoreductive surgery | Primary cytoreductive surgery  Secondary cytoreductive surgery  Interval cytoreductive surgery  hyperthermic intraperitoneal chemotherapy OR HIPEC | (Homologous recombination proficiency OR HRP) AND (primary cytoreductive surgery OR PCS) AND ovarian cancer  (Homologous recombination proficiency OR HRP) AND (secondary cytoreductive surgery OR SCS) AND ovarian cancer  (Homologous recombination proficiency OR HRP) AND (hyperthermic intraperitoneal chemotherapy OR HIPEC) AND ovarian cancer |
| Chemotherapy | Platinum-based  Chemosensitivity  Platinum re-treatment  Dose-dense | (Homologous recombination proficiency OR HRP) AND chemotherapy  Chemotherapy AND PARP inhibitor AND ovarian cancer |
| Antiangiogenic treatment | VEGF  Bevacizumab  Cediranib | Bevacizumab AND maintenance AND ovarian cancer  VEGFR3 AND PARP inhibitor AND ovarian cancer  EGFR PARPi OC |
| PARPi | PARP inhibitor  Niraparib  Rucaparib | PARP inhibitor AND (Homologous recombination proficiency OR HRP)  PARP inhibitor AND maintenance AND ovarian cancer  PARP inhibitor AND resistance AND ovarian cancer  PARP inhibitor AND immune checkpoint inhibitor AND ovarian cancer |
| Immunotherapy | Mirvetuximab  Antibody-drug conjugate  Tumor infiltrating lymphocytes OR TILs  Peritumoral T cells  Mutational burden  Immune checkpoint blockade  Immune excluded  Immune desert  Cold tumors  T cell infiltrations  Gemogenovatucel-T  Vigil  Adoptive cell therapy | (Homologous recombination proficiency OR HRP) AND (Tumor infiltrating lymphocites OR TILs)  (Homologous recombination proficiency OR HRP) AND microenvironment  (Homologous recombination proficiency OR HRP) AND immunotherapy  (Homologous recombination proficiency OR HRP) AND (Antibody drug conjugate OR ADC) AND ovarian cancer  (Homologous recombination proficiency OR HRP) AND immune checkpoint AND ovarian cancer  (Homologous recombination proficiency OR HRP) AND vigil  (Homologous recombination proficiency OR HRP) AND (Adoptive cell therapy OR ACT) AND ovarian cancer |
| Combined therapies | Combined therapies (homologous recombination proficiency OR HRP)  CDK inhibitor  WEE1 inhibitor  PKMYT1  ATR inhibitor  Pl3K/AKT inhibitor  Pl3K inhibitor  AKT inhibitor  HDAC inhibitor  HSP90 inhibitor  BET inhibitor | CDK inhibitor AND PARP inhibitor AND ovarian cancer  WEE1inhibitor AND PARP inhibitor AND ovarian cancer  PKMYT1 AND ATR inhibitor AND ovarian cancer  PKMYT1 AND chemotherapy AND ovarian cancer  ATR inhibitor AND PARP inhibitor AND ovarian cancer  Pl3K/AKT inhibitor AND PAPR inhibitor AND ovarian cancer  HDAC inhibitor AND PARP inhibitor AND ovarian cancer  HSP90 inhibitor AND PARP inhibitor AND ovarian cancer  BET inhibitor AND PARP inhibitor AND ovarian cancer |
